# Anterograde monosynaptic transneuronal tracers derived from herpes simplex virus 1 strain H129

**DOI:** 10.1186/s13024-017-0179-7

**Published:** 2017-05-12

**Authors:** Wen-Bo Zeng, Hai-Fei Jiang, Ya-Dong Gang, Yi-Ge Song, Zhang-Zhou Shen, Hong Yang, Xiao Dong, Yong-Lu Tian, Rong-Jun Ni, Yaping Liu, Na Tang, Xinyan Li, Xuan Jiang, Ding Gao, Michelle Androulakis, Xiao-Bin He, Hui-Min Xia, Ying-Zi Ming, Youming Lu, Jiang-Ning Zhou, Chen Zhang, Xue-Shan Xia, Yousheng Shu, Shao-Qun Zeng, Fuqiang Xu, Fei Zhao, Min-Hua Luo

**Affiliations:** 1 State Key Laboratory of Virology, CAS Center for Excellence in Brain Science and Intelligence Technology (CEBSIT), Wuhan Institute of Virology, Chinese Academy of Sciences, Wuhan, 430071 China; 2 University of Chinese Academy of Sciences, Beijing, 100049 China; 3 Britton Chance Center for Biomedical Photonics, Wuhan National Laboratory for Optoelectronics-Huazhong University of Science and Technology, Wuhan, 430074 China; 4 State Key Laboratory of Membrane Biology, School of Life Sciences; PKU-IDG/McGovern Institute for Brain Research, Peking University, Beijing, 100871 China; 5 Chinese Academy of Science Key Laboratory of Brain Function and Diseases, School of Life Sciences, University of Science and Technology of China, Hefei, 230027 China; 6 State Key Laboratory of Cognitive Neuroscience and Learning, IDG/McGovern Institute for Brain Research, School of Brain and Cognitive Sciences, the Collaborative Innovation Center for Brain Science, Beijing Normal University, Beijing, 100875 China; 7 Department of Physiology, School of Basic Medicine and Tongji Medical College; The Institute for Brain Research, Collaborative Innovation Center for Brain Science, Huazhong University of Science and Technology, Wuhan, 430030 China; 8 Guangzhou Women and Children’s Medical Center, Guangzhou Medical University, Guangzhou, 510623 China; 9 Department of Neurology, School of Medicine, University of South Carolina, Columbia, SC 29203 USA; 10 State Key Laboratory of Magnetic Resonance and Atomic and Molecular Physics, Brain Research Center, Wuhan Institute of Physics and Mathematics, Chinese Academy of Sciences, Wuhan, 430071 China; 11 The 3rd Xiangya Hospital, Central-South University, Changsha, 410013 China; 12 Faculty of Life Science and Technology, Kunming University of Science and Technology, Kunming, 650500 China

**Keywords:** Herpes simplex virus type 1 (HSV-1), H129 strain, Anterograde, Neuronal tracer, H129, ΔTK, tdT, Monosynaptic, H129-G4, Multisynaptic

## Abstract

**Background:**

Herpes simplex virus type 1 strain 129 (H129) has represented a promising anterograde neuronal circuit tracing tool, which complements the existing retrograde tracers. However, the current H129 derived tracers are multisynaptic, neither bright enough to label the details of neurons nor capable of determining direct projection targets as monosynaptic tracer.

**Methods:**

Based on the bacterial artificial chromosome of H129, we have generated a serial of recombinant viruses for neuronal circuit tracing. Among them, H129-G4 was obtained by inserting binary tandemly connected GFP cassettes into the H129 genome, and H129-ΔTK-tdT was obtained by deleting the thymidine kinase (TK) gene and adding tdTomato coding gene to the H129 genome. Then the obtained viral tracers were tested in vitro and in vivo for the tracing capacity.

**Results:**

H129-G4 is capable of transmitting through multiple synapses, labeling the neurons by green florescent protein, and visualizing the morphological details of the labeled neurons. H129-ΔTK-tdT neither replicates nor spreads in neurons alone, but transmits to and labels the postsynaptic neurons with tdTomato in the presence of complementary expressed TK from a helper virus. H129-ΔTK-tdT is also capable to map the direct projectome of the specific neuron type in the given brain regions in Cre transgenic mice. In the tested brain regions where circuits are well known, the H129-ΔTK-tdT tracing patterns are consistent with the previous results.

**Conclusions:**

With the assistance of the helper virus complimentarily expressing TK, H129-ΔTK-tdT replicates in the initially infected neuron, transmits anterogradely through one synapse, and labeled the postsynaptic neurons with tdTomato. The H129-ΔTK-tdT anterograde monosynaptic tracing system offers a useful tool for mapping the direct output in neuronal circuitry. H129-G4 is an anterograde multisynaptic tracer with a labeling signal strong enough to display the details of neuron morphology.

**Electronic supplementary material:**

The online version of this article (doi:10.1186/s13024-017-0179-7) contains supplementary material, which is available to authorized users.

## Background

Mapping brain connectome is essential for understanding how the brain works, and mapping the damage of brain connectome is critical for understanding the mechanism(s) for neurodegenerative brain disorders, including Alzheimer disease (AD) and Parkinson disease (PD). As the basic unit of neural function, neuronal circuit serves as the bridge between macroscale (structure/function) and microscale (molecules/signal pathways). Viral tracers have contributed to discovery of novel circuits and revealing new features of known canonical circuits. Efficiently mapping the neuronal circuits requires both retrograde and anterograde tracers transmitting multi- or monosynaptically. Rabies virus (RV) and Pseudorabies virus (PRV) derived viral tools represent the retrograde mono- and multisynaptic tracers to map the input neural networks [1]. While, herpes simplex virus type 1 (HSV-1) strain 129 (H129) is a promising anterograde multisynaptic neuronal circuit tracer [2, 3].

HSV-1 is a ubiquitous opportunistic pathogen, establishing latency in ganglion post primary infection, usually harmless in immunocompetent individuals, but frequently causing cold sores, occasionally sporadic encephalitis in immunocompromised individuals [4]. The natural neuronal tropism and transneuronal spread capacity make it a promising neuronal circuit tracer. HSV-1 strains have preference in transneuronal transmission, among which McIntyre-B spreads retrogradely, whereas strain H129 displays anterograde transneuronal transmission [4–6]. Multiple studies have employed H129 for multisynaptically mapping the projection circuits and output networks in different animal models [4, 7–10]. In particular, the development of genetically modified H129 recombinant virus that expresses fluorescent protein made it possible to directly visualize the output neural pathways, and greatly prompted the application of H129 tracer [11, 12]. Besides H129, vesicular stomatitis virus (VSV) was also reported for anterograde circuit tracing [13, 14]. While these multisynaptic anterograde tracers are useful for mapping the output neural pathways [11–14], anterograde monosynaptic tracers to determine the direct or indirect projection targets are urgently required.

In the present study, we introduce a potential novel anterograde monosynaptic tracer H129-ΔTK-tdT and a bright anterograde multisynaptic tracer H129-G4. With helper virus complementarily expressing TK, H129-ΔTK-tdT can potentially transmit to the postsynaptic neurons, and enable visualization of direct projection targets of either a given brain nucleus or a specific neuron type. H129-G4 may label the multisynaptic projection circuit with high labeling intensify, which helps to visualize the details of neuron morphology along the circuits. These novel anterograde tracers offer novel tools for projectome mapping, and complement the existing neuronal circuit tracing tool box.

## Results

### Labeling efficiency of H129

**Fig. 1:**
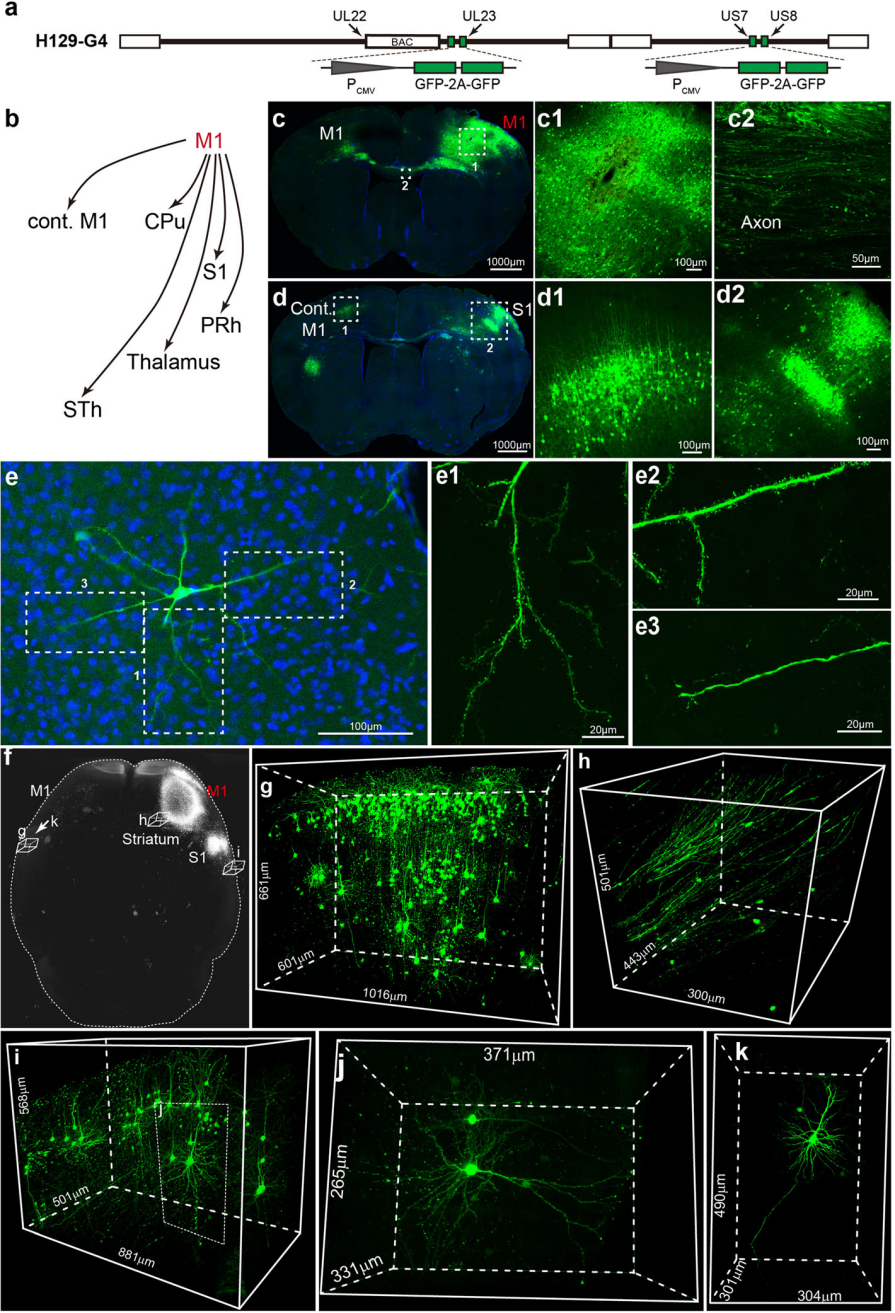
H129 derived tracer H129-G4 (**a**) The schematic diagram of H129-G4 genome. Two binary GFP elements were inserted into the genome of H129 bacterial artificial chromosome (H129-BAC) at the indicated position. **b** Schema of the simplified M1 projection pathways. The M1 pathway has been simplified and only the representative M1 projection targets are displayed. M1, primary motor cortex; cont. M1, contralateral M1; S1, primary somatosensory cortex; PRh, perirhinal cortex; STh, subthalamic nucleus; CPu, caudate putamen. **c**–**d** Representative tracing result of H129-G4. H129-G4 was injected into M1 of wild-type C57BL/6 mice, and representative images of coronal brain sections were obtained at 4 days post infection (dpi). Representative regions are shown, and the boxed areas are presented in the right panels with a higher magnification. **e** A representative H129-G4 labeled single neuron. A representative GFP-labeled neuron in PRh is shown, and the magnified images of the dendritic segments with individual spines (e1 and e2) and the axon (e3) are presented in the right panels. **f**–**k** Combination of fMOST and H129-G4 tracing. The mouse brain obtained at 4 dpi was further processed to fMOST imaging. The 3D image of the whole brain was reconstructed (**f**). Representative brain regions, including the contralateral S1 (**g**), striatum (**h**) and the ipsilateral S1 (**i**), are shown in details. Representative single neuron images at the ipsilateral S1 (**j**) and the contralateral S1 (**k**) are also presented

To examine the labeling efficiency of H129, we constructed a replication competent recombinant virus H129-G4 that contains 4 GFP genes in the genome (Fig. 1a). H129-G4 is capable to spread transneuronally through the primary motor cortex (M1) pathway and efficiently label the downstream brain regions of M1 (Fig. 1b-d). The fluorescence intensity of H129-G4 is sufficient to clearly label and visualize the neuron morphological details, including the dendrites, spines and axonal fibers (Fig. 1e). The bright labeling makes H129-G4 compatible with the fluorescence Micro-Optical Sectioning Tomography (fMOST) (Fig. 1f-k) [15], while other versions containing 1 to 3 copies of GFP genes created by us, as well as the H129 tracers from Lo and McGovern [11, 12], failed to combine with fMOST due to insufficient labeling intensity (data not shown). The combination of H129-G4 tracing and fMOST imaging enables the high-throughput imaging and automated reconstruction of neuronal circuit in the entire brain with high resolution at submicron levels.

In addition, H129-G4 is also applicable to trace the neuronal circuit of the tree shrew (*Tupaia belangeri chinensis*) (Additional file 1: Figure S1), a small mammal more closely related to the primates than rodents at behavioral, anatomical, genomic, and evolutionary levels [16, 17]. These results indicate a high tracing efficiency of H129-G4.

### Invasion and transmission of H129

Next, we employed microfluidic plates to characterize the invasion and transmission properties of H129 in vitro [18]. The plate consists of two isolated culture chambers connected by multiple microchannels (Additional file 2: Figure S2a), which only allows the grow-through of axons but impedes that of dendrites (Additional file 2: Figure S2b). Presynaptic marker synaptophysin and postsynaptic density protein (PSD-95) were observed in the afferent chamber, suggesting the potential synapse formation near the microchannels (Additional file 2: Figure S2c). Notably, the hydrostatic pressure generated from the medium height difference guarantees the flow direction in the microchannel and completely prevents the diffusion or leakage of the virus to the opposite chamber (Additional file 2: Figure S2d) [18]. The replication competent tracer H129-G4, which serve as a reference for the mono-synaptic tracer H129-ΔTK-tdT described below, was applied to the microfluidic plate to investigate its in vitro invasion and transmission property.

**Fig. 2:**
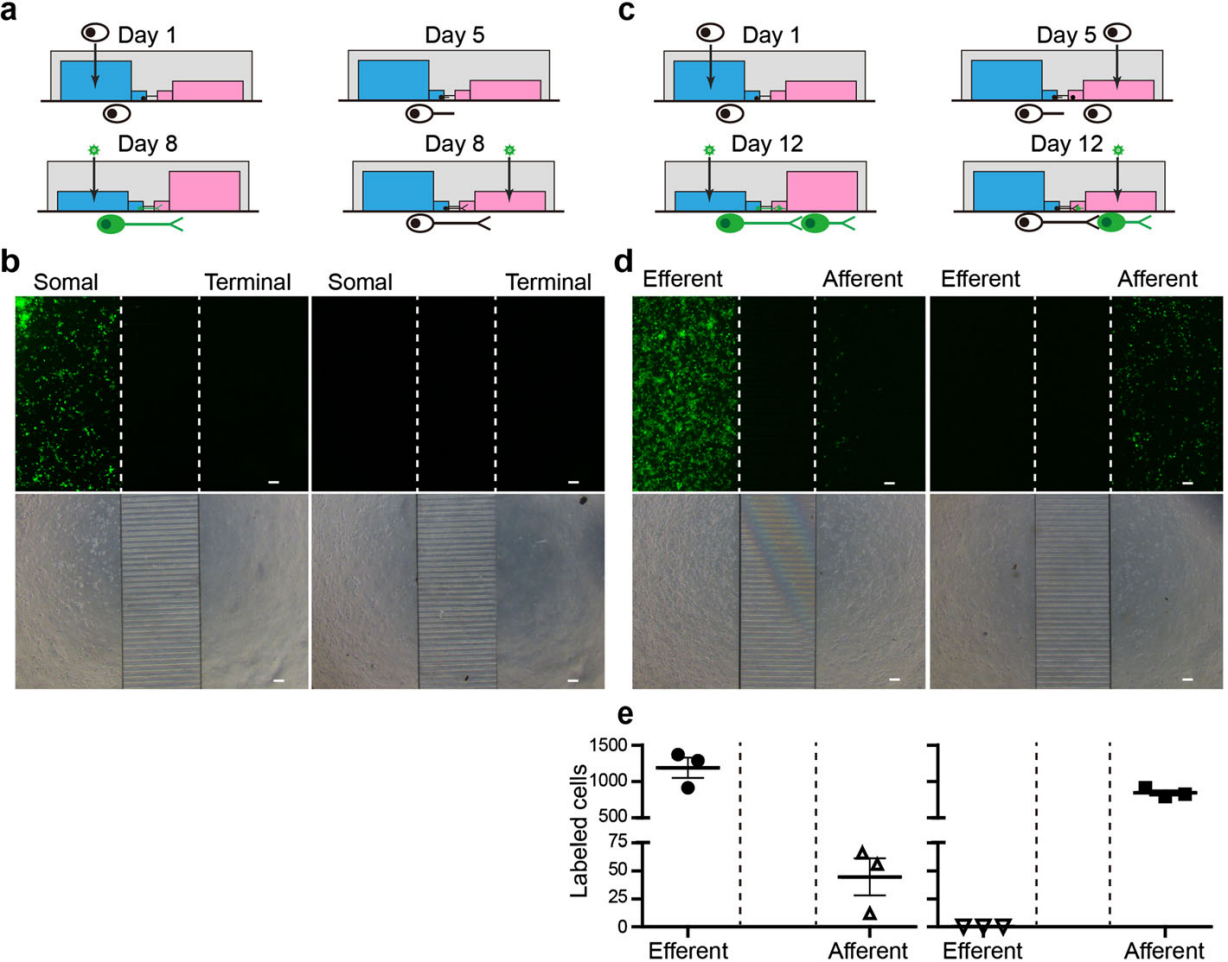
Invasion and transmission of H129-G4 in vitro (**a**–**b**) Invasion of H129-G4. Freshly isolated fetal mouse hippocampal and cortical neurons were seeded into one chamber of the microfluidic plate, and the first 24 h culture was termed as Day 1. H129-G4 was added at Day 8 into either the soma (blue) or axon terminal chamber (red) to a final concentration of 1 × 10^7^ pfu/ml (**a**). Images of GFP signal (upper panel) and phase contrast (lower panel) were obtained at 24 h post infection (hpi). The represent results from 3 microchannel plates are shown (**b**). The dotted lines indicate the borders between chambers and the microchannels. Scale bar = 100 μm (**c**–**e**) Transmission direction of H129-G4. Neurons were sequentially plated into the two chambers at Day 1 and Day 5, then H129-G4 was added at Day 12 into either the efferent (blue) or afferent chamber (red) to a final concentration of 1 × 10^7^pfu/ml (**c**). Images of GFP signal (upper panel) and phase contrast (lower panel) were obtained at 24hpi. The representative results from 3 microfluidic plates are shown (**d**), and the quantitative analysis were performed by counting the GFP labeled cell mounts in different chambers (**e**). The dotted lines indicate the borders between chambers and the microchannel. Scale bar = 100 μm

To determine the virus invasion feature, mouse hippocampal and cortical neurons cultured in one chamber, and H129-G4 was added to either the soma or axon terminal side (Fig. 2a). H129-G4 in the soma side well labeled the neurons with GFP by 24 h post infection (hpi) (Fig. 2b, left panel). When H129-G4 was added to the axonal terminal side to a final concentration of 1 × 10^7^ pfu/ml, no GFP labeled neuron was observed by 24 hpi (Fig. 2b, right panel) and even up to 96 hpi (data not shown). On the contrary, VSV labelled a vast number of somas by terminal invasion at the same concentration. (Additional file 3: Figure S3c). Terminal invasion of H129 has been reported previously [2], which was also observed in our microfluidic assay with higher H129-G4 concentrations and extended infection time (Additional file 3: Figure S3a-b). The positive correlation between the terminal invasion incidence and virus concentration suggests the necessity of optimizing the injection virus dose for in vivo tracing. Notably, H129-G4 displayed greater retrograde labeling incidence in ganglion neurons than in hippocampal and cortical neurons, suggesting that terminal invasion varies in different types of neuronal cells (Additional file 3: Figure S3b).

To test the transneuronal transmission direction in vitro, neurons were plated in both chambers with a 4-day interval between culture initiation in each chamber (Fig. 2c). H129-G4 was added to either the efferent or afferent chamber to a final concentration of 1 × 10^7^ pfu/ml, a dose causing no observed terminal invasion (Fig. 2c). During infection in efferent neurons, H129-G4 spread through the axons in the microchannels, and labeled the afferent neurons in the opposite chamber (Fig. 2d, left panel). But H129-G4 in the afferent chamber only efficiently infected the afferent neurons, and no GFP labeled efferent neuron was observed at 24 hpi (Fig. 2d, right panel) and even up to 72 hpi (data not shown). The fluorescence intensity in the chambers were quantified, and no retrograde transmission were observed in all experiments (Fig. 2e). These data indicate that H129-G4 transmits between the cultured neurons in the microfluidic plate in an anterograde manner.

Since the microchannel system can’t completely mimic the in vivo physiological condition, we further determined the invasion and transmission feature of H129 in vivo.

The unidirectional projection from retina to the lateral geniculate nucleus (LGN) provides an ideal pathway to investigate the terminal invasion and transmission direction of H129 in vivo [19]. In consistence with previous reports [2, 6, 11], H129-G4 efficiently transmitted anterogradely from the retina, labeled LGN and downstream brain regions (Additional file 4: Figure S4). LGN directly projects to layer IV of primary visual cortex (V1) [20], and GFP signal was indeed observed at this layer when H129-G4 was injected in to LGN. But retinas were also labeled when H129-G4 was injected to LGN, indicating terminal invasion to retinas and/or the viruses released from retinas secondarily infecting retina cells (Additional file 5: Figure S5a). The labeled retina cell number and their occurrence time are correlated with the dose of injected virus, again suggesting the importance of optimizing tracing condition and setting parallel retrograde tracer control in vivo. No retrograde transmission was observed under this applied experimental condition, as H129-G4 injected at V1 didn’t spread to retinas through LGN (Additional file 5: Figure S5b). Some non-ganglion cells in the retina were GFP positive when H129-G4 was injected into LGN, especially with higher dose of virus, which was probably due to the secondary infection of virus progeny released from the dead infected ganglion cells. It is of significance to test the retrograde transsynaptic transmission of H129 with the monosynaptic tracer H129-ΔTK-tdT, since the replication competent H129-G4 may cause secondary infection by the offspring virus released from infected neurons.

To investigate H129H transmission feature, H129H-G4 was also examined in the well documented neural circuit between ventral posteromedial thalamic nucleus (VPM) and the primary somatosensory cortex (S1) (Additional file 6: Figure S6a) [21–23]. H129-G4 was injected into the VPM together with Alexa Fluor 594 conjugated-cholera toxin B subunit (CTB), which labels the soma of the upstream neuron via axon terminal absorption. GFP labeled neurons were sequentially observed at nRT (2 dpi, Additional file 6: Figure S6b) and layer 4 of S1 cortex (4 dpi, Additional file 6: Figure S6c). Notably, the GFP-positive neurons and CTB-labeled neurons formed two segregated cell populations located in layer 4 and 6 of S1 at 4dpi, respectively, and no overlap of the two populations was observed (Additional file 6: Figure S6c). These data indicate that H129-G4 anterogradely and transsynaptically transmits from VPM to layer 4 of S1, but does not label layer 6 neurons via axon terminals in the VPM.

Taken together, H129 mainly infects neurons through the soma and transmits to postsynaptic neurons anterogradely. However, terminal invasion of H129 was observed in cultured neurons and retina ganglia cells, whose efficiency is correlated with virus dose.

### The anterograde monosynaptic tracer H129-ΔTK-tdT

**Fig. 3:**
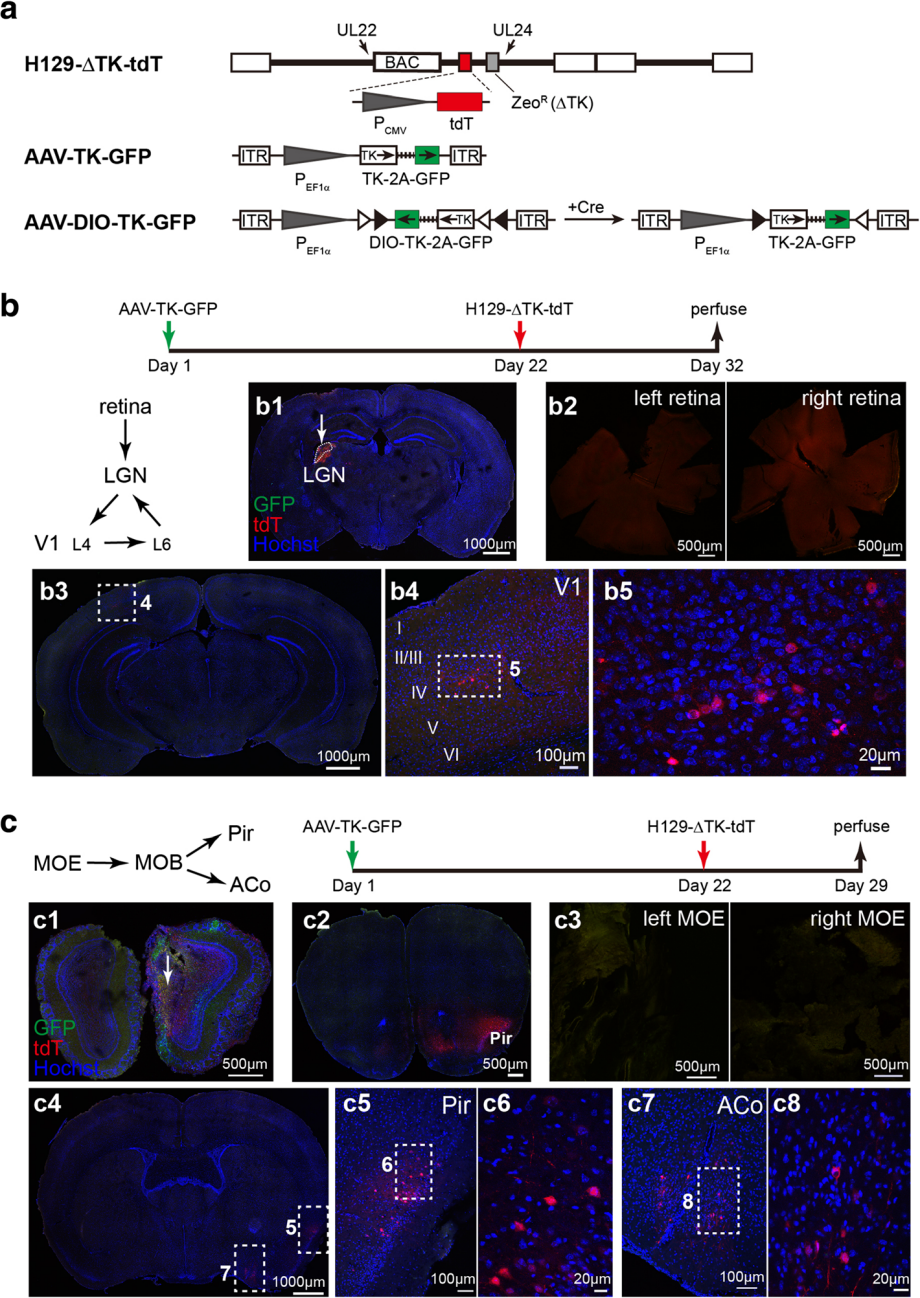
In vivo transmission of H129-ΔTK-tdT (**a**) Schematic genome diagrams of H129-ΔTK-tdT and the helper viruses. H129-ΔTK-tdT was generated by replacing the thymidine kinase gene (TK, UL23) of H129-BAC with CMV promoter controlled tdTomato (tdT) gene and the selection marker of zeocin resistance gene (Zeo^R^). The helper viruses of AAV-TK-GFP and AAV-DIO-TK-GFP express TK and GFP under the control of EF1α promoter either in the Cre-independent or -dependent manner, respectively. **b**–**c** Transmission of H129-ΔTK-tdT tracing system in vivo. AAV-TK-GFP and H129-ΔTK-tdT were sequentially injected into LGN (**b**) and MOB (**c**) with the dose following the table described in Materials and methods. Representative upstream or downstream brain regions were examined as shown in the simplified connectivity paths. The representative results from 3 mice per group are shown. LGN, lateral geniculate nucleus; V1 (L4, L6), layer 4, layer 6 of primary visual cortex, MOE, main olfactory epithelium; MOB, main olfactory bulb; Pir, piriform; ACo, anterior cortical amygdaloid nucleus

H129-ΔTK-tdT was generated by knocking out TK gene (Fig. 3a). Consistent with previous report [24], TK deficiency caused mild growth delay of H129 in Vero cells, but impaired viral replication in cultured fetal mouse neurons (Additional file 7: Figure S7). With helper virus AAV-TK-GFP or AAV-DIO-TK-GFP compensatorily expressing TK (Fig. 3a), H129-ΔTK-tdT transmits one step to the postsynaptic neuron.

We examined the in vivo transmission direction of this monosynaptic tracing system in the visual and olfactory pathways [19, 25, 26]. AAV-TK-GFP and H129-ΔTK-tdT were injected to LGN or the main olfactory bulb (MOB) as shown in the schematics (Fig. 3b and c). Neurons labeled with tdTomato were observed in the regions of the injection sites (LGN and MOB) (Fig. 3b1 and c1), and the downstream regions including cortical layer IV of V1 (transmitted from LGN) (Fig. 3b3–5), piriform cortex (Pir) (Fig. 3c2 and 4–7) and anterior cortical amygdaloid nucleus (ACo, transmitted from MOB) (Fig. 3c7–8), indicating the anterograde transmission of H129-ΔTK-tdT. No tdTomato-labeled neuron at the corresponding upstream regions (retina or the main olfactory epithelium, MOE) was observed (Fig. 3b2 and c3), suggesting no retrograde labeling of H129-ΔTK-tdT, including both terminal invasion and retrograde transmission, under the experimental condition. Different from H129-G4, H129-ΔTK-tdT didn’t label the retinas and MOE neurons via terminal invasion, probably because of the optimized injection dose and deficient replication (Materials and methods Tables 1 and 2). When H129-ΔTK-tdT was injected to these two regions alone, tdTomato-positive cells outside the injection sites were not observed (Additional file 8: Figure S8f and b).

**Fig. 4:**
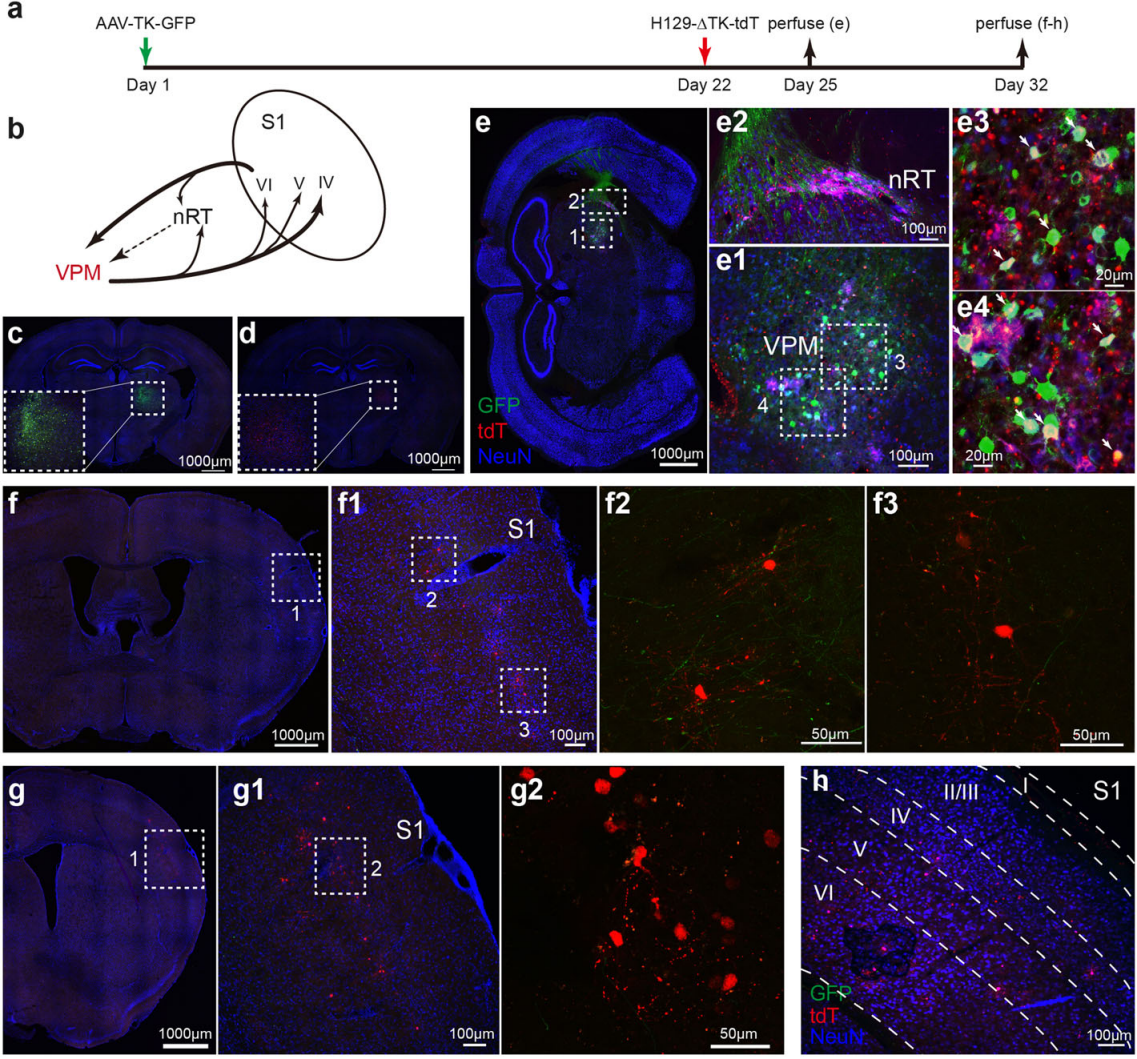
Tracing VPM pathway with H129-ΔTK-tdT (**a**) Experiment time line for tracing VPM pathway with H129-ΔTK-tdT and the helper. **b** Schema of the simplified VPM-S1 circuit. The projection of the inhibitory interneurons at nRT was indicated by the dotted line. VPM, ventral posteromedial thalamic nucleus; nRT, nucleus of reticular thalamus; S1, primary somatosensory cortex; IV, V and VI, layer 4, 5 and 6 of the cortex. **c**–**d** Controls of the helper and H129-ΔTK-tdT alone. AAV-TK-GFP (**c**) or H129-ΔTK-tdT (**d**) was individually injected into the VMP of wild-type C57BL/6, and images were obtained at 21 (**c**) or 10 dpi (**d**), respectively. The injection sites are shown in the dotted boxes. **e** The starter neurons of H129-ΔTK-tdT transmission. AAV-TK-GFP and H129-ΔTK-tdT were injected into VPM of wild-type C57BL/6 mice at Day 1 and 22 sequentially. Brains were obtained at Day 25, and coronal brain slices were stained with anti-NeuN and anti-tdTomato antibodies. The magnified image of the injection site (e1) and nRT (e2) is shown, and the representative regions are further magnified (e3 and e4). The starter neurons, which expresses both tdTomato and GFP, are indicated with the white arrows. **f**–**h** Tracing the monosynaptic output of VPM. The animals were perfused at Day 32, and the coronal brain slices throughout the brains were observed. The representative tracing images of the S1 (**f**–**h**) in VPM circuit are shown. The cortical layers at S1 are determined according to NeuN staining and indicated by the dotted lines (**h**)

**Fig. 5:**
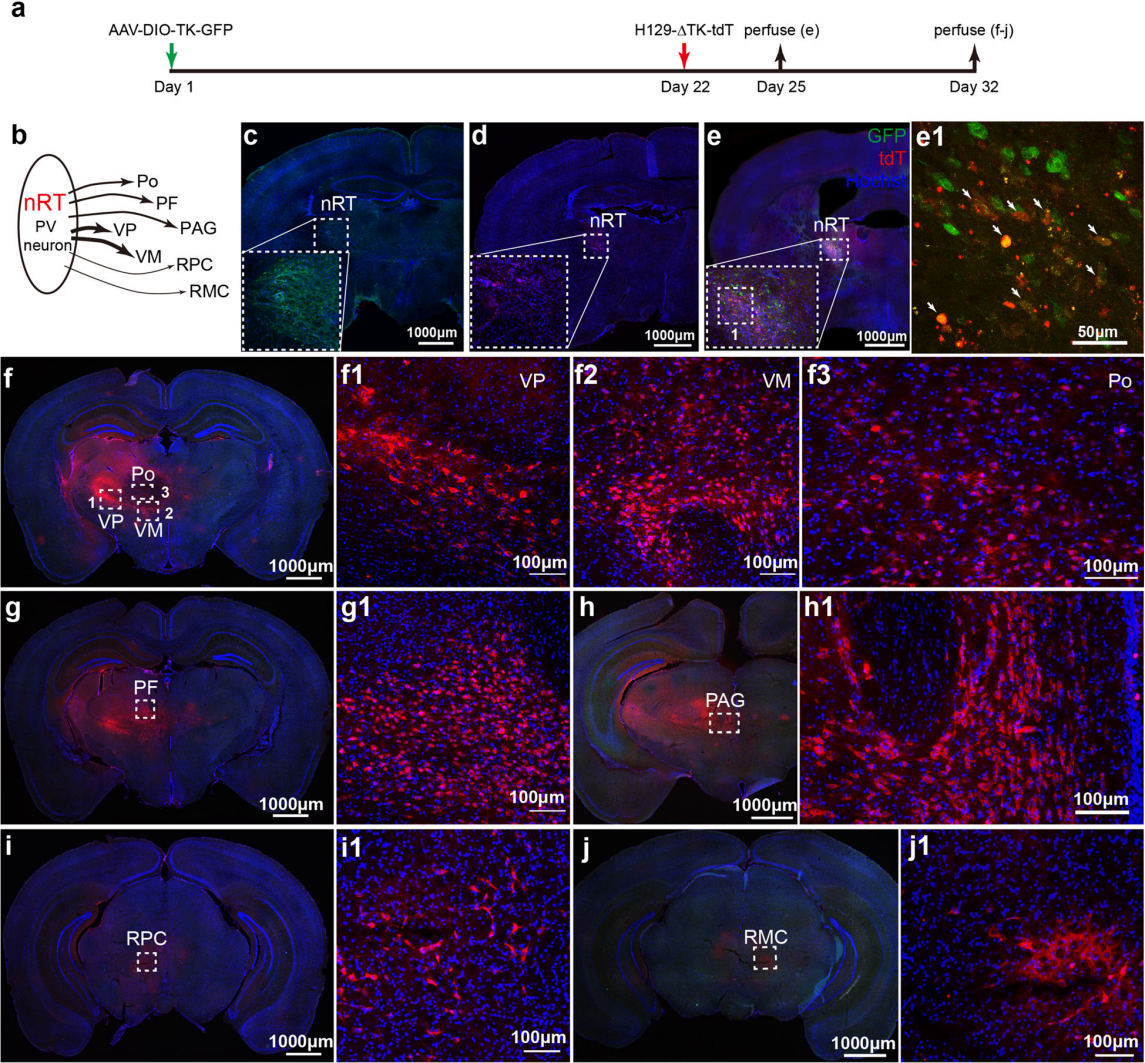
Mapping the direct projections from nRT-PV neurons with H129-ΔTK-tdT. **a** Experiment time line for tracing the VPM pathway with H129-ΔTK-tdT and the helper. **b** Schema of the direct projections from PV neuron at nRT. PV neuron, parvalbumin neuron; nRT, thalamic reticular nucleus; VP, ventral posterior nucleus; VM, Ventral medial nucleus; Po, posterior thalamic nuclear group; PF, parafascicular thalamic nucleus; PAG, periaqueductal gray; RPC, red nucleus, parvicellular part; RMC, red nucleus, magnocellular part. **c**–**d** Controls of the helper and H129-ΔTK-tdT alone. AAV-DIO-TK-GFP (c) or H129-ΔTK-tdT (**d**) was individually injected into the nRT of PV-Cre mice, and the images were obtained at 21 (**c**) and 10 dpi (**d**), respectively. The injection sites are shown with the dotted boxes. (**e**) The starter neurons of H129-ΔTK-tdT transmission. AAV-DIO-TK-GFP and H129-ΔTK-tdT were injected into nRT of PV-Cre mice at Day 1 and 22 sequentially, and images were obtained at Day 25. The image of the injection site at nRT is shown (**e**), and the representative region is further magnified (e1). The starter neurons, which express both tdTomato and GFP, are indicated with the white arrows. **f**–**j** Tracing the monosynaptic output of nRT-PV neurons. The animals were perfused at Day 32, and the coronal brain slices throughout the entire brains were observed. Shown are the representative images labeled by H129-ΔTK-tdT, including VP, VM, Po (**f**), PF (**g**), PAG (**h**), RPC (**i**) and RMC (**j**). Selected regions indicated with dotted boxes are further magnified correspondingly

**Table 1:** Experimental parameters for H129-G4 tracing

Animal	Nucleus	Coordinates (mm)			Dose (pfu)	Vol. (μl)	Perfusion time	Animalnumber
		ML	AP	DV			(dpi)	
C57BL/6	M1	-1.70	+1.54	−1.75	1.0 × 10^6^	0.2	4	3
C57BL/6	retina	NA	NA	NA	5.0 × 10^6^	1.0	4	4
C57BL/6	LGN	+2.13	−2.30	−2.75	5.0 × 10^4^ / 5.0 × 10^5^	0.1	3 and 5	4
C57BL/6	V1	+2.30	−3.52	−1.15	5.0 × 10^4^ / 5.0 × 10^5^	0.1	4 and 8	4
C57BL/6	VPM	+1.40	−1.82	−3.62	1.0 × 10^6^	0.2	2–5	8
Tree shrew	M1	−2.60	+1.85	−2.10	2.0 × 10^6^	0.3	6	5

**Table 2:** Experimental parameters for monosynaptic tracing

Mouse	Nucleus	Coordinate (mm)			Helper virus (1 × 10^12^ v.g./ml)			H129-ΔTK-tdT			Examination time (Day)		Animalamount
		ML	AP	DV	Type	Vol. (μl)	Injection (Day)	Dose (pfu)	Vol. (μl)	Injection (Day)	Starterneuron	Postsynapticneuron	
C57BL/6	MOB	-0.48	+4.28	-2.30	AAV-TK-GFP	0.45	Day 1	1.00 × 10^5^	0.40	Day 22	25	29	4
C57BL/6	LGN	+2.13	−2.30	-2.75	AAV-TK-GFP	0.20	Day 1	1.00 × 10^5^	0.20	Day 22	25	32	4
C57BL/6	VPM	-1.42	−1.82	-3.55	AAV-TK-GFP	0.20	Day 1	1.00 × 10^5^	0.20	Day 22	25	32	5
PV-Cre	nRT	+1.50	−0.82	-3.75	AAV-DIO-TK-GFP	0.35	Day 1	1.25 × 10^5^	0.25	Day 22	25	32	5
DAT-Cre	VTA	−0.40	−3.28	-4.30	AAV-DIO-TK-GFP	0.35	Day 1	1.25 × 10^5^	0.25	Day 22	25	32	5

To further examine the terminal invasion and infection tropism in the central nervous system (CNS), H129-ΔTK-tdT was injected into more brain regions (Additional file 8: Figure S8). Terminal invasion was not detected in 5 of 6 tested regions (Additional file 8: Figure S8b-g), except the only case in CA1. A few neurons in LEC, an upstream nucleus of CA1, were labeled by H129-ΔTK-tdT, indicating that terminal invasion may occur in certain brain regions (Additional file 7: Figure S7e). In all the regions tested, H129-ΔTK-tdT displayed neuron tropism, and no virus-infected glia (GFAP positive) was observed, since all tdTomato-positive cells were NeuN positive but GFAP negative (Additional file 8: Figure S8b-g).

These results show that the H129-ΔTK-tdT is a monosynaptic tracer which anterogradely transmits and labels the postsynaptic neurons. However, proper controls are strongly recommended to exclude the misleading labeling caused by the potential terminal invasion,

### Monosynaptic labeling of VPM output using H129-ΔTK-tdT

Next, the H129-ΔTK-tdT monosynaptic tracing system was tested in the well documented neuronal circuit between the ventral posteromedial thalamic nucleus (VPM) and the primary somatosensory cortex (S1) [21–23, 27]. Neurons at VPM send their axons to S1 and form synaptic contacts in cortical layer IV, V and VI; projection neurons in layer VI in turn project backward to VPM and the nucleus of reticular thalamus (nRT). Reciprocal connections also occur locally in the thalamus between relay neurons and GABAergic neurons in nRT (Fig. 4b).

AAV-TK-GFP and H129-ΔTK-tdT were injected into the VPM of wild-type C57BL/6 mice in sequence (Fig. 4a). Similar to AAV-TK-GFP alone, injection of H129-ΔTK-tdT alone only weakly labeled neurons around the injection sites but not the connected regions, indicating no terminal invasion nor non-specific transmission occurred under this condition (Fig. 4c and d). When both viruses were injected to the same site at VPM sequentially (Day 1 and Day 22), neurons expressing both GFP and tdTomato were observed at the injection site at Day 25 (Fig. 4e). These double-labeled neurons were the starter cells, in which AAV-TK-GFP complementally expressed TK supporting H129-ΔTK-tdT replication and production of virus progeny. An average of 163.0 ± 21.83 starter cells were observed in three mice (Additional file 9: Figure S9a). The reproduced viral tracers anterogradely transmitted to the postsynaptic neurons and labeled them with tdTomato. However, due to the deficient viral replication by lack of TK, H129-ΔTK-tdT was restrained in the postsynaptic neurons. The defect of viral replication resulted in weak tdTomato expression, thus antibody staining was required to amplify the labeling signal for visualization.

A few nRT neurons expressed tdTomato were first observed at Day 25 (Fig. 4e2). At Day 32, tdTomato labeled neurons were then observed at various VPM innervating regions including cortical layer IV, V, VI of S1 (Fig. 4f-h). The quantitative analysis of the labeled neuron’s amount were performed and presented (Additional file 9: Figure S9a). The axonal fibers originated from the VPM could be visualized by AAV expressed GFP (Fig. 4f2 and f3). Notably, H129-ΔTK-tdT labeled neurons in nRT appeared earlier than those in S1, probably caused by, at least partially, the difference in projection distances from VPM to these two regions.

### Cre dependent anterograde monosynaptic tracing in transgenic mice

Precisely mapping the output neuronal circuits required not only the output information from given brain regions, but also the projectome paths from specific type of neurons. For this purpose, H129-ΔTK-tdT was applied with AAV-DIO-TK-GFP helper in appropriate Cre-transgenic mice. Under the control of the Double-floxed Inverted Orientation (DIO) Cre-On system, AAV-DIO-TK-GFP expresses TK and GFP only in the presence of Cre recombinase (Fig. 3a), which assists H129-ΔTK-tdT monosynaptic transmission specifically from the Cre expressing neurons [28].

PV-Cre transgenic mice specifically express Cre recombinase in parvalbumin (PV) interneurons. The nRT contains abundant PV-neurons, which receive inputs from cerebral cortex and dorsal thalamus and project widely back to thalamic nucleuses to regulate the flow of information from thalamus to cortex (Fig. 5b) [29, 30]. So nRT was chosen as the virus injection site. Consistent with previous results, AAV-DIO-TK-GFP and H129-ΔTK-tdT restrictedly labeled the neurons around the injection site by itself (Fig. 5c-d). When both viruses were injected sequentially, GFP and tdTomato co-expressing neurons were observed at nRT at Day 25 (Fig. 5e). Since AAV-DIO-TK-GFP expresses GFP and TK specifically in Cre neurons, the yellow neurons were superinfected by both AAV-DIO-TK-GFP and H129-ΔTK-tdT, and represented the starters which initiated viral transmission. At Day 32, neurons expressing tdTomato were observed in various brain regions, including ventral posterior nucleus (VP), ventral medial nucleus (VM), posterior thalamic nuclear group (Po), parafascicular thalamic nucleus (PF), periaqueductal gray (PAG), red nucleus parvicellular part (RPC) and red nucleus magnocellular part (RMC) (Fig. 5f-j). The starter neurons and transsynaptically labeled neurons were counted, and the data were shown in Additional file 9: Figure S9b. Importantly, no neurons in the S1 was labeled, indicating absence of axon terminal invasion and retrograde transmission (Additional file 10: Figure S10).

To rule out the possibility that the nucleuses adjacent to nRT were labeled due to the virus diffusion instead of the transneuronal transmission, this system was further validated by tracing long distance projection.

**Fig. 6:**
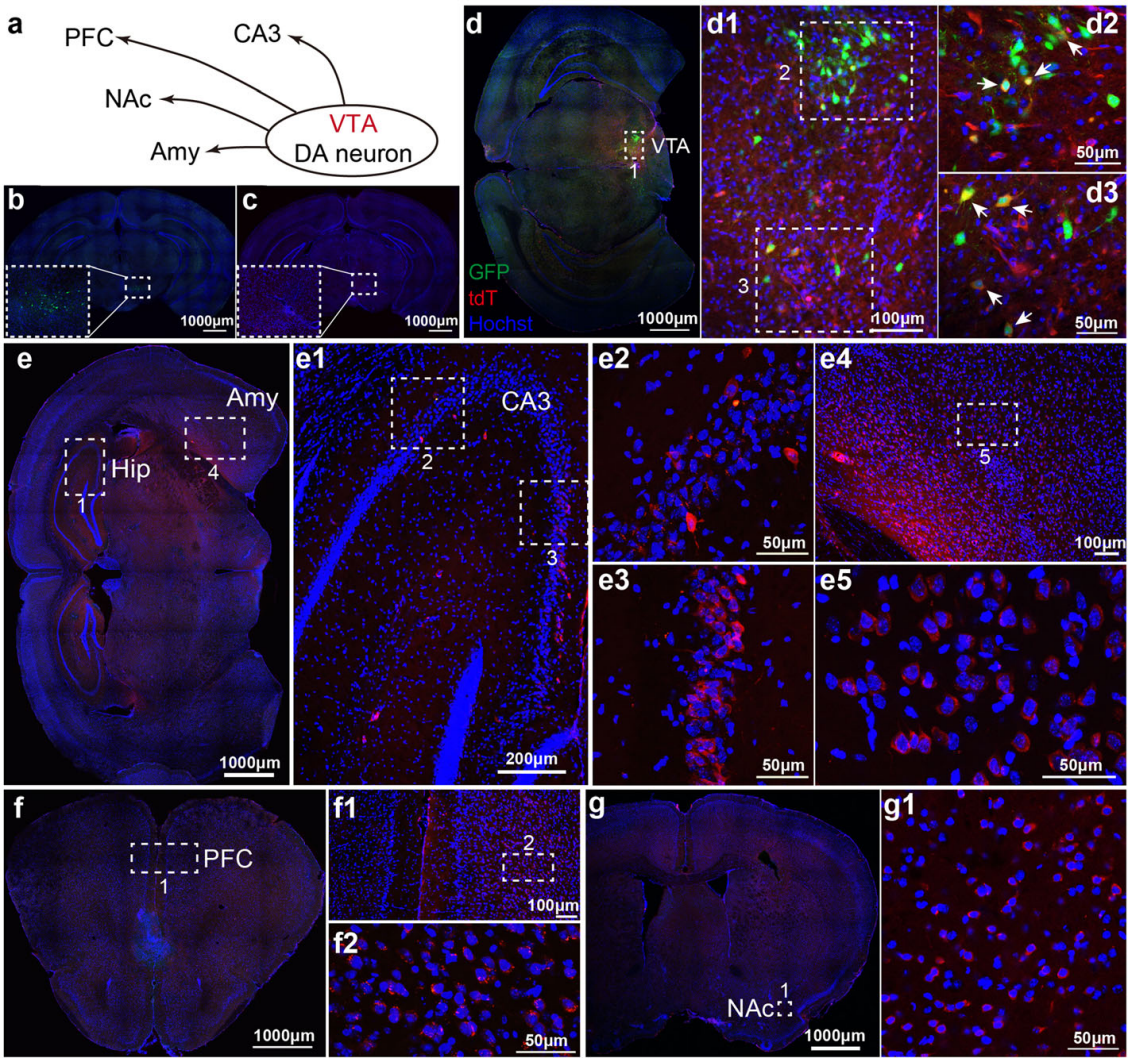
Mapping the direct projections from VTA-DA neurons with H129-ΔTK-tdT. **a** Schema of the direct projections from DA neurons at VTA. DA neuron, dopaminergic neuron; VTA, ventral tegmental area; CA3, Hippocampus CA3; Amy, amygdala; NAc, nucleus accumbens; PFC, prefrontal cortex. **b**–**c** Controls, the helper and H129-ΔTK-tdT alone. AAV-DIO-TK-GFP (**b**) and H129-ΔTK-tdT (**c**) was individually injected into the VTA of DAT-Cre mice, and the images were obtained at 21 (**b**) and 10 dpi (**c**), respectively. The injection sites are shown in the dotted boxes. **d** The starter neurons of H129-ΔTK-tdT transmission. AAV-DIO-TK-GFP and H129-ΔTK-tdT were injected into the VTA of DTA-Cre mice at Day 1 and 22 sequentially, and images were obtained at Day 25. The image of the injection site at VTA is shown (d1), and the representative regions are further presented with a higher magnification (d1-d3). The starter neurons, which express both tdTomato and GFP, are indicated with the white arrows. **e**–**g** Tracing the monosynaptic output of VTA-DA neurons. The animals were perfused at Day 32, and the coronal brain slices throughout the entire brains were observed. Representative images of VTA-DA neuron innervating regions, including hippocampus (e1–3), amygdala (e4–5), PFC (**f**) and NAc (**g**), are presented, and the boxed regions are further magnified

DAT-Cre transgenic mice express the Cre recombinase in dopaminergic (DA) neurons under the control of the dopamine transporter (DAT) promoter. DA neurons in ventral tegmental area (VTA) widely implicate in the brain reward pathway and directly project to hippocampus, prefrontal cortex (PFC), nucleus accumbens (NAc) and amygdala (Fig. 6a) [31–33]. Neurons labeled by individually injected viruses were restrained to the area around the injection site at VTA (Fig. 6b and c), which is consistent with the results described above. Starter neurons were observed at VTA at Day 25 (Fig. 6d). At Day 32, tdTomato labeled neurons in the Hipp-CA3, amygdala, NAc and as far as PFC, indicated that H129-ΔTK-tdT successfully transsynaptically spread from the starter DA neurons to these nuclei (Fig. 6e-g). The quantitative analysis was performed as presented (Additional file 9: Figure S9c).

Taken together, H129-ΔTK-tdT with the help of AAV-DIO-TK-GFP can monosynaptically trace the direct anterograde connections from a specific type of neurons expressing Cre.

## Discussion

Damage of brain connectome in neurodegenerative diseases remains unclear. Revealing the differences and abnormalities of neuronal circuits between healthy individuals and patients with PD or AD will contribute to understanding the mechanism(s) of these diseases. Mapping the brain connectome requires appropriate tracing tools, but the anterograde tracing tools are underdevelopment, and particularly, the monosynaptic anterograde tracer is still lacking.

Thymidine kinase (TK) synthesizes thymidine monophosphate (TMP) by catalyzing thymidine and ATP, which is an essential step for thymidine triphosphate (TTP) synthesis [34]. Most proliferating cells, including Vero, express the cellular TK, which compensate the viral TK deficiency and enable viral replication. However, none or limited cellular TK catalytic in non-proliferating cells, such as neuron, leads insufficient TTP, and further impairs the virus genome synthesis. Therefore H129-ΔTK-tdT alone doesn’t spread among neurons in vivo. When administrated together, the coinfected AAV helper complimentarily expresses TK, and supports H129-ΔTK-tdT genome replication, leading the viral proteins synthesis and viral particle assembly. Then the newly propagated viral particles transmit through the axons, travel across the synapses, reach the postsynaptic neurons in the same way as the wild type H129 particle, and express tdTomato to label the cells. Therefore, the anterograde monosynaptic tracing potential of H129-ΔTK-tdT is achieved and guaranteed by the *in cis* deletion and *in trans* complementation of viral TK.

With the rich repertoire of Cre transgenic mouse lines, the H129-ΔTK-tdT anterograde monosynaptic tracing system offers a possibility to map the direct projectome of a specific type of neuron in a given brain region. When mapping the direct projectome using H129-ΔTK-tdT and the helper, it is always necessary to optimize the experimental conditions, such as applying the parallel control of H129-ΔTK-tdT injection without AAV helper to preclude potential terminal invasion, carefully choosing the proper H129-ΔTK-tdT injection dose to obtain ideal labeling efficiency (normally 100–300 nl of 3–5 × 10^8^ pfu/ml virus), and perfusing at the appropriate time points post H129-ΔTK-tdT injection for desired mapping observation (normally from 3 to 12 days).

We also developed H129-G4, an efficient anterograde multisynaptic tracer with Strong GFP labeling. Enhancement of labeling intensity is an useful improvement for clearly visualizing the details of projection pathway and neuron morphology including dendrites, spines, and axonal fibers. The intensity improvement also expands the potential utility of H129-G4, such as imaging with fMOST, which contributes to map whole-brain connectome with high resolution in a high-throughput and automated way.

Besides the predominant anterograde transmission, H129 and its derivatives also infect nerve terminals and undergo retrograde transport to the cell body [2, 7, 8, 11, 35], which were also observed in our study both in vitro and in vivo. From the careful examinations, our data showed that terminal invasion is observed at higher doses and extended observation time. The incidence of terminal invasion is correlated with the virus dose, neuron type and brain region, suggesting the importance of optimization the experiment conditions. The microfluidic plate applied in our study has a slow but sustained medium flow in the microchannel to counteract diffusion [18]. It guarantees that neither Dextran [18] nor viruses leaks to the opposite chamber. The cell type tropism might be another feature for H129 terminal invasion, since in ganglion neurons H129-G4 showed a higher terminal invasion incidence than in neurons isolated from the brain. Hematopoietic stem cell expansion: challenges and opportunities published data described the release of HSV from neurons [36], and infectious H129-G4 virus particle have also been detected in the supernatant of infected neurons in our own lab. So the “retrograde transmission” H129 reported in some publication was possibly caused by secondary infection instead of genuine transneuronal retrograde spreading [2]. Thus, it is more accurate to examine the H129 transmission direction using the monosynaptic tracing system as shown.

The relative short and unpredictable survival time of the injected animals is a limitation of multisynaptic H129 tracers [11, 37, 38], which is also observed with H129-G4. This shortage is overcome in the H129-ΔTK-tdT monosynaptic tracing system. TK deficiency impairs the viral replication in neurons and thus significantly attenuates its animal toxicity by limiting its spreading. Either upon injection alone or sequentially with AAV helper, no virus induced animal death was observed up to 20 dpi at the dose of 5 × 10^5^ pfu H129-ΔTK-tdT. The mice displayed none or only mild symptoms.

High cytotoxicity of the replication competent H129 brings obstacle to the functional analysis. Although the replication of H129-ΔTK-tdT is severely impaired, very low levels of the viral proteins still present in the infected neurons, which mildly impact the normal cellular function. TK complemented by AAV helper restores H129-ΔTK-tdT replication to a certain level, and boosts level of viral proteins, which leads to severer cell damage than H129-ΔTK-tdT alone. The cytopathic effects induced by TK deficient virus is attenuated compared to H129-G4. The shortages of inducing cytopathy and the requirement for signal enhancement by antibody staining limit the application of H129-ΔTK-tdT for direct functional tracing, such as electrophysiology, optogenetic assay, and calcium imaging etc. But H129-ΔTK-tdT is still capable of identifying potential postsynaptic targets and facilitates the identification of the cell types, which could be further verified by independent functional assays.

## Conclusion

We have created the anterograde multi- and monosynaptic tracers derived from HSV-1 H129 strain. H129-G4 is a multisynaptic tracer with labeling intensity strong enough to label the details of neuron morphology. H129-ΔTK-tdT represents a potential novel anterograde monosynaptic tracer, which may contribute in revealing the direct projectome connectivity. These tracers complement the current neuronal circuit tracer tool box.

## Methods

### Ethics statement

The standards of performance (SOP) and animal studies have been approved by the Institutional Review Board and Institutional Animal Welfare Committee (WIVA10201502), including neuron isolation and intracerebral inoculation of mice and tree shrews with viral tracers. All the experiments with viruses were performed in bio-safety level 2 (BSL-2) laboratory and animal facilities.

### Cells and cell culture

Vero-E6 cell (Vero, ATCC#CRL-1586) was purchased from ATCC, maintained our own laboratory and tested to be Mycoplasma free. The culture medium is Dulbecco’s modified Eagle medium (DMEM, Cat. #12100046, Gibco/Life technologies) containing 10% fetal bovine serum (FBS, Cat. #12483020, Gibco/Life technologies) and penicillin-streptomycin (100 U/ml of penicillin and 100 μg/ml of streptomycin, Cat. #15140122, Gibco/Life technologies).

According to the protocol described previously [39–42], hippocampal and cortical neurons were isolated from the forebrain of C57BL/6 mouse pups at embryonic day 18.5 (E18.5), and ganglion neurons were isolated from dorsal root ganglion (DRG) or trigeminal ganglion (TRG) of 4-week male C57BL/6 mice. Briefly, the cerebral cortex together with hippocampus were dissociated with trypsin (Cat. #15400054, Gibco/Life technologies)/DNase I (Cat. #D5025, Sigma) for 15 min at 37 °C. Isolated neurons were washed with Hank’s Balance Salt Solution (HBSS) without calcium and magnesium (Cat. #14170112, Gibco/Life technologies), resuspended and cultured in Neurobasal medium (Cat. #21103049, Gibco/Life technologies) supplemented with 2% B27 (Cat. #17504044, Gibco/Life technologies), 25 μM GlutaMAX (Cat. #35050079, Gibco/Life technologies) and penicillin-streptomycin. Medium was changed every other day. DRG and TRG dissection were treated with papain (Cat. #P4762, Sigma) solution for 15 min, then with collagenase (Cat. #17101015, Gibco/Life technologies)/dispase (D4693, Sigma) solution for addition 15 min, both at 37 °C. Percoll (Cat. #17–0891-01, GE Healthcare) gradient centrifugation was applied to separate myelin and nerve debris from sensory neurons. Acquired neurons were cultured with Neurobasal medium supplemented with 50 ng/ml mouse beta-nerve growth factor (β-NGF, Cat. #cyt-581, ProSpec), 2% B27, GlutaMAX and penicillin-streptomycin.

### Construction of the recombinant H129 tracers and helper viruses

Both of H129-G4 and H129-ΔTK-tdT were derived from the H129 bacterial artificial chromosome (H129-BAC). H129-BAC was constructed by inserting the F-plasmid vector pUS-F5 into the H129 genome between UL22 and UL23 by homologous recombination as described previously [43]. Two binary GFP elements were inserted into H129-BAC sequentially at the indicated sites to generate H129-G4 (Fig. 1a). H129-ΔTK-tdT and H129-tdT were obtained by inserting tdTomato genes (tdT) and the Zeocin resistant gene (Zeo^R^) into H129-BAC with or without deleting thymidine kinase (TK) gene (UL23) respectively (Fig. 3a). The viral genome manipulations were performed in *E. coli* DY380 strain via homologous recombination, and validated by PCR and sequencing. The recombinant viruses were reconstituted and produced in Vero cells following the protocol described previously [43, 44]. The average viral titer after concentrating were about 2–5 × 10^9^ pfu/ml for H129-G4 and 5–10 × 10^8^ pfu/ml for H129-ΔTK-tdT.

Two helper viruses, AAV-TK-GFP and AAV-DIO-TK-GFP were constructed using the vector of adeno-associated virus serotype 9, and were packaged by the Obio Technology Corporation (Shanghai, China). AAV-TK-GFP constitutively expresses TK and GFP, supporting H129-ΔTK-tdT monosynaptically transmission. AAV-DIO-TK-GFP conditionally expresses TK and GFP only in the presence of the Cre recombinase (Fig. 2a).

### Fluorescence micro-optical sectioning tomography (fMOST)

Specimen for fMOST imaging was embedded with Technovit 9100 Methyl Methacrylate (MMA, Electron Microscopy Sciences) using an optimized protocol to preserve the fine structures labeled with GFP. Briefly, PFA fixed animal brain was rinsed in 0.01 M PBS for 12 h, and completely dehydrated in a series of alcohol (50%, 75%, 95%, 100% and 100% ethanol, 2 h for each) followed by immersion in xylene twice (2 h for each) for transparentization. After dehydration, the brain was ready for infiltration: it was successively soaked in a graded series of infiltration solutions (50%, 75%, 100% and 100% resin in 100% ethanol, 2 h each for the first three solutions and 48 h for the final solution), Then the specimen was transferred into gelatin capsule and immersed in polymerization solution. Finally, the capsule with the specimen was closed and kept in a dry chamber at -4 °C in dark for 72 h. After complete polymerization, the resin-embedded mouse brain was immersed into 0.05 M Na_2_CO_3_ buffer to enhanced the GFP signal. With real time reactivating fluorescence, the whole brain was imaged using fMOST system at 0.5 μm × 0.5 μm × 1 μm voxel size [45, 46]. Lastly, the image stack of the acquired data set was transformed into Large Data Access using the Amira software (Visage Software, San Diego, CA, USA) for 3D image reconstruction [47].

### Microfluidic assay

The microfluidic plates were fabricated following the protocol described previously [18, 40, 48]. The microchannel mask and chamber mask were designed accordingly and produced by Microclear Electronics Technology (Nanjing, China). Photoresist SU-8 GM 1050 and GM 1075 (Gersteltec Sarl), propylene glycol methyl ether acetate (PGMEA, Sigma), poly (dimethylsiloxane) (PDMS) (Sylgard 184, Dow Corning) were used to fabricate the microfluidic devices. The microchannels of the plate are 700 μm length, 10 μm width and 3 μm depth (Additional file 2: Figure S2a).

To culture neurons in the microfluidic plate, freshly isolated neurons (5 × 10^5^ of fetal mouse cortical and hippocampal neurons, and 1 × 10^5^ of ganglion neurons) were plated into one chamber and cultured in 400 μl medium (Day 1) (blue chamber in Fig. 2a and c). For bi-chamber culture, a new batch of neurons (1 × 10^5^ neurons) were added into the opposite chamber (red chamber in Fig. 2c) and cultured in 200 μl medium at Day 5 of initiation of neurons culture, when the axons of the first plated neurons had grew into the microchannels. Medium was refreshed every day, and the volume in the chamber was maintained at 400 μl and 200 μl to generate the hydrostatic pressure. H129-G4 was added into the chamber with negative hydrostatic pressure to avoid virus diffusion to the opposite chamber, and the medium volume was changed accordingly prior to infection.

When indicated, the neurons grown on microfluidic plate were stained. The primary antibodies included rabbit anti-MAP2 polyclonal antibody (Cat. #17490–1-AP, Proteintech), mouse monoclonal antibody of anti-Tau (IgG1, Cat. #ab80579, Abcam), −SYP (IgG1, Cat. #sc-17,750 SantaCruz), −PSD95 (IgG2a, Cat. #75–028, NeuroMab). The secondary antibodies included Alexa Fluor 488 conjugate Goat anti-Rabbit IgG (H + L) (Cat. #A-11008, Invitrogen), Alexa Fluor 594 conjugate Goat anti-Mouse IgG1 (Cat. #A-21125, Invitrogen), Alexa Fluor 488 conjugate Goat anti-Mouse IgG1 (Cat. #A-21121, Invitrogen), and Alexa Fluor 594 conjugate Goat anti-Mouse IgG2a (Cat. #A-21135, Invitrogen).

For quality control, a few microfluidic plates from each fabrication batch (50 plates) were randomly selected to check the inter-compartment leakage. 5 × 10^4^ Vero cells were cultured in one chamber for 5 days, and H129-G4 was added to the opposite chamber (2.5 × 10^9^ pfu/ml) with less medium volume and lower hydrostatic pressure (Additional file 2: Figure S2d). GFP signals were daily monitored up to 3dpi. The batch of microfluidic plates were applied for experiment only when no leakage was observed in all tested plates.

### Intracerebral virus injection

Intracerebral virus injection was performed using a stereotaxic system in a BSL-2 animal facility following the approved SOP on 8 week-old male mice or adult tree shrews without randomization or blinding. The mice included wild-type, PV-Cre and DAT-Cre transgenic C57BL/6 mice. DAT-Cre mice specifically express Cre recombinase in dopaminergic (DA) neurons under the control of the dopamine transporter (DAT) promoter, and PV-Cre mice express Cre recombinase in parvalbumin (PV) interneurons. The anesthetized animals received intracerebral virus injection with a motorized stereotaxic injector (Stoelting Co.). The exact parameters of the mouse nucleus location was determined according to the Mouse Brain Atlas by the mediolateral (ML), anteroposterior (AP) and dorsoventral (DV) distances to Bregma [49].

H129-G4 injection was performed as listed in Table 1. When indicated, Alexa Fluor 594-conjugate cholera toxin subunit B (CTB, Cat. #C22842, Molecular Probes) was injected together with the virus to mark the injection site. Animals were anesthetized and perfused with sterile normal saline and 4% paraformaldehyde (PFA) solution at the indicated time points. The whole brain was carefully removed, fixed with 4% PFA, dehydrated in 30% sucrose, and sectioned at -20 °C for imaging. Animals were monitored daily after the virus injection, and experiment would be terminated and animal would be excluded if severe sickness was observed.

For monosynaptic tracing, helper viruses and H129-ΔTK-tdT were injected at Day 1 and 22 respectively. Perfusion were performed on Day 25 to 32. The helper viruses and H129-ΔTK-tdT were also individually injected to the same site as controls. The detailed injection parameters are listed in Table 2.

### Examination of the neural circuitry tracing

The obtained brains were coronally cryo-sectioned to 40 μm thickness slices using a microtome (HM550, Thermo/Life technologies). Neurons were stained with rabbit anti-NeuN (Cat. #ab104225, Abcam) and Alexa Fluor 647-conjugated goat anti-rabbit antibodies (Cat. #A-21245, Thermo/Life technologies) when indicated. Otherwise, the cell nuclei were counterstained with Hoechst dye 33,342 (Cat. #H3570, Thermo/Life technologies). All images were obtained using a Nikon’s A1R MP+ confocal microscope equipped with a fast high resolution galvanometer scanner. The tdTomato signal was amplified by staining with rabbit anti-DsRed polyclonal antibody (Cat. #632496, Takara) and Alexa Fluor 594-conjugated goat anti-rabbit IgG (H + L) (Cat. #A-11037, Invitrogen). When stained with NeuN, mouse anti-tdTomato monoclonal antibody (IgG2b, Cat. #TA180009, Origene) and the secondary antibody of Alexa Fluor 594-conjugate goat anti-mouse IgG2b (Invitrogen, Cat. #A-21145) were used.

## Additional files

Additional file 1: Figure S1. Application of H129-G4 in tree shrew (a) Comparison of mouse and tree shrew brains. The brains of adult mouse and tree shrew are imaged with top (left) or side view (middle) after perfusion and fixation. The average size and weight of the brains are presented (right) as mean ± SD (standard deviation) from 5 animals in each group. (b-g) Tracing results H129-G4 in tree threw M1 circuit. H129-G4 and CTB were injected into the M1of adult tree shrews, and the brains were perfused at 6 dpi. Representative images of the coronal brain sections are presented, and the boxed regions are displayed with a higher magnification. M1, primary motor cortex; IRd, infraradiata dorsalis; Pir, piriform cortex; Pu, putamen; Cl, claustrum (Cl); PC, paracentral thalamic nucleus; VL, ventrolateral thalamic nucleus; V1, primary visual cortex. (h) A representative H129-G4 labeled single neuron in tree threw. A representative GFP-labeled neuron around the injection site is shown, and the magnified images of the apical (h1-h3) and basal dendrites (h4) are presented in the right panels. (PDF 3120 kb)

Additional file 2: Figure S2. The microfluidic plate (a) The schematic structure diagram of the microfluidic system. (b) Axons through the microchannels. Freshly isolated fetal mouse hippocampal and cortical neurons were seeded into one chamber of the microfluidic plate, and cultured for 7 days with positive hydrostatic pressure in the soma chamber. Then the plate was disassembled and stained with antibodies against Tau and Map2. Shown is the representative image from 3 plates. (c) Pre- and post-synaptic markers in the afferent chamber. Neurons were sequentially plated into both chambers at Day 1 and Day 5 respectively, and cultured for additional 7 days with positive hydrostatic pressure in the efferent chamber. The plate was disassembled on Day 12 and stained for pre-synaptic marker synaptophysin (SYP) and post-synaptic marker PSD-95. The nuclei were counterstain with Hoechst dye. Shown is the representative image from 3 plates. (d) No inter-compartment leakage between the chambers. Vero cells were cultured in one chamber with positive hydrostatic pressure, and H129-G4 was added into the opposite chamber to a final concentration of 2.5 × 10^9^ pfu/ml. The GFP signal in the Vero cell culture chamber was monitored, and show is the representative image at 72 hpi. (PDF 676 kb)

Additional file 3: Figure S3. Terminal invasion of H129-G4 in vitro (a) Dose related incidence of H129-G4 terminal invasion. Fetal mouse hippocampal and cortical neurons were cultured as described above, H129-G4 was added to the terminal chamber at the indicated final concentrations, and GFP signal was monitored daily. Representative images at 48 hpi from 3 plates at each concentration group are shown. The labeled neurons are indicated with the dotted boxes and magnified in the lower panels. Scale bar = 100 μm. (b) Census of H129-G4 terminal invasion. Hippocampal and cortical neurons, trigeminal ganglion (TG) and dorsal root ganglion neurons (DRG) were cultured in microfluidic plates at the indicated cell amount. H129-G4 was added to the axonal terminal chamber at different final concentrations, and the GFP positive neurons in each plate were counted. Data were from 3 plates under each condition, and results are presented as mean ± SD. (c) Terminal invasion of VSV. Similarly, VSV-GFP was added into the terminal chamber of hippocampal and cortical neurons to a final concentration of 1 × 10^7^ pfu/ml. The representative image from 3 plates at 48 hpi is shown. Scale bar = 100 μm. (PDF 429 kb)

Additional file 4: Figure S4. Anterograde transmission of H129-G4 from retina to CNS (a) Schema of the simplified mouse visual pathway. LGN, lateral geniculate nucleus; LP, lateral posterior thalamic nucleus; SC, superior colliculus; V1 and V2, primary and secondary visual cortex. (b-d) H129-G4 tracing from retina. H129-G4 was injected into the right retina of wild-type C57BL/6 mice, and images were obtained at 6 dpi. Representative images at LGN, LP (b) and visual cortex (c) are shown. Selected regions are magnified correspondingly, and representative single neurons are presented (d). (PDF 1100 kb)

Additional file 5: Figure S5. Invasion and transmission of H129-G4 in the visual pathway. Different amount of H129-G4 was injected into the left LGN (a) or V1 (b) of wild-type C57BL/6 mice together with CTB, respectively. The animals were perfused at the indicated time points, and the coronal brain slices throughout the entire brains were observed. Representative images at V1, LGN and retinas are presented. (PDF 1400 kb)

Additional file 6: Figure S6. Invasion and transmission of H129-G4 in the VPM-S1 circuit. (a) Simplified schema of the VPM-S1 circuit. VPM, ventral posteromedial thalamic nucleus; nRT, nucleus of reticular thalamus; S1, primary somatosensory cortex; IV, V and VI, layer 4, 5 and 6 of the cortex. (b) Representative tracing results of H129-G4 in VPM-S1 circuit. H129-G4 (1 × 10^6^ pfu in 200 nl) was injected into the VPM of wild-type C57BL/6 mice together with Alexa Fluor 594-conjugated CTB (CTB, red). The animals were perfused at the indicated time points, and representative images of the coronal brain slice at the VPM-S1 regions are shown. The boxed areas are magnified and presented in the right panel. The layers of the S1 cortex were determined according to NeuN staining and indicated by the dotted lines. (PDF 503 kb)

Additional file 7: Figure S7. In vitro replication of the H129-ΔTK-tdT. To determine the growth property of H129-ΔTK-tdT which lacks TK, fetal mouse hippocampal and cortical neurons (Neuron) or Vero cells were infected with H129-ΔTK-tdT or the TK competent strain H129-tdT at an MOI of 0.02. At the indicated time point, virus titers in the cell culture were determined by standard plaque forming assay. Shown is the representative data from 3 independent experiments, and presented as the Mean ± SD from triplicates. (PDF 183 kb)

Additional file 8: Figure S8. H129-ΔTK-tdT alone in various brain regions. (a) Parameters for H129-ΔTK-tdT injection. (b-g) Representative labeling results. H129-ΔTK-tdT was injected into the indicated brain regions of wild type C57BL/6 as listed in the table (a), and the brains were perfused at 10 dpi. The coronal brain slice throughout the brain were observed after staining for NeuN (blue), GFAP (green) and tdTomato (red). Representative images of upstream and downstream region of the injection sites are shown. The white arrows indicated the injection sites. The neurons in the upstream brain region labeled by virus terminal invasion are indicated by the white arrowheads (e3). OB, olfactory bulb; Pir, piriform cortex; MOE, main olfactory epithelium; M1, primary motor cortex; DG, dentate gyrus; LEC, lateral entorhinal cortex; LGN, lateral geniculate nucleus; V1, primary visual cortex; VTA, ventral tegmental area; LDTg, laterodorsal tegmental nucleus. (PDF 2370 kb)

Additional file 9: Figure S9. Quantitative analysis of H129-ΔTK-tdT monosynaptic tracing. Helper AAVs and H129-ΔTK-tdT were sequentially injected at VPM (a), nRT (b) or VTA (c) of wild type C57BL/6, PV-Cre or DAT-Cre mice respectively, as described in Fig.4-6. The brains’ corona slices were observed with an interval of 120 μM (every 4 slices), and the amounts of the labeled cell at the indicated brain regions were counted. The starter cells at the injection site were detected at Day32 as tdTomato and GFP co-expressing neurons (opened circle, each circle represents one mouse), and the tdTomato positive neurons at other brain regions were observed at Day 32 (filled triangle, each circle represents one mouse). (PDF 109 kb)

Additional file 10: Figure S10. Absence of labeled cells upstream of nRT-PV neurons. H129-ΔTK-tdT and AAV-DIO-TK-GFP were injected into the nRT of PV-Cre mice as shown in Fig.5. The animals were perfused Day 32, and the coronal brain slices throughout the entire brains were observed. The representative images of S1, the upstream region of nRT, are shown. (PDF 929 kb)

## Abbreviations

ACo: Anterior cortical amygdaloid nucleus
CA1: Field CA1 of hippocampus
CNS: Central nervous system
Cre: Cre recombinase
CTB: Cholera toxin B subunit
DA: Dopamine
DAT: Dopamine transporter
DIO: Double-floxed inverted orientation
fMOST: The fluorescence micro-optical sectioning tomography
GABA: Gamma-aminobutyric acid
GFAP: Glial fibrillary acidic protein
GFP: Green fluorescent protein
H129: Herpes simplex virus type 1 strain 129
hpi: Hour post infection
HSV-1: Herpes simplex virus type 1
LGN: Lateral geniculate nucleus
M1: Primary motor cortex
MOB: Main olfactory bulb
MOE: Main olfactory epithelium
NAc: Nucleus accumbens
NeuN: Neuronal nuclear antigen
NRT: Reticular thalamus
PAG: Periaqueductal gray
PF: Parafascicular thalamic nucleus
PFC: Prefrontal cortex
pfu: Plaque forming unit
Pir: Piriform cortex
Po: Posterior thalamic nuclear group
PV: Parvalbumin
RMC: Red nucleus magnocellular part
RPC: Red nucleus parvicellular part
RV: Rabies virus
S1: Primary somatosensory cortex
tdT: Tdtomato
TK: Thymidine kinase
V1: Primary visual cortex
VM: Ventral medial nucleus
VP: Ventral posterior nucleus
VPM: Ventral posteromedial thalamic nucleus
VSV: Vesicular stomatitis virus
VTA: Ventral tegmental area
