# Supplementary figures and images for: Anterograde monosynaptic transneuronal tracers derived from herpes simplex virus 1 strain H129
Source: Mol Neurodegener. 2017 May 12;12:38. doi: 10.1186/s13024-017-0179-7 (PMC5427628; doi:10.1186/s13024-017-0179-7)

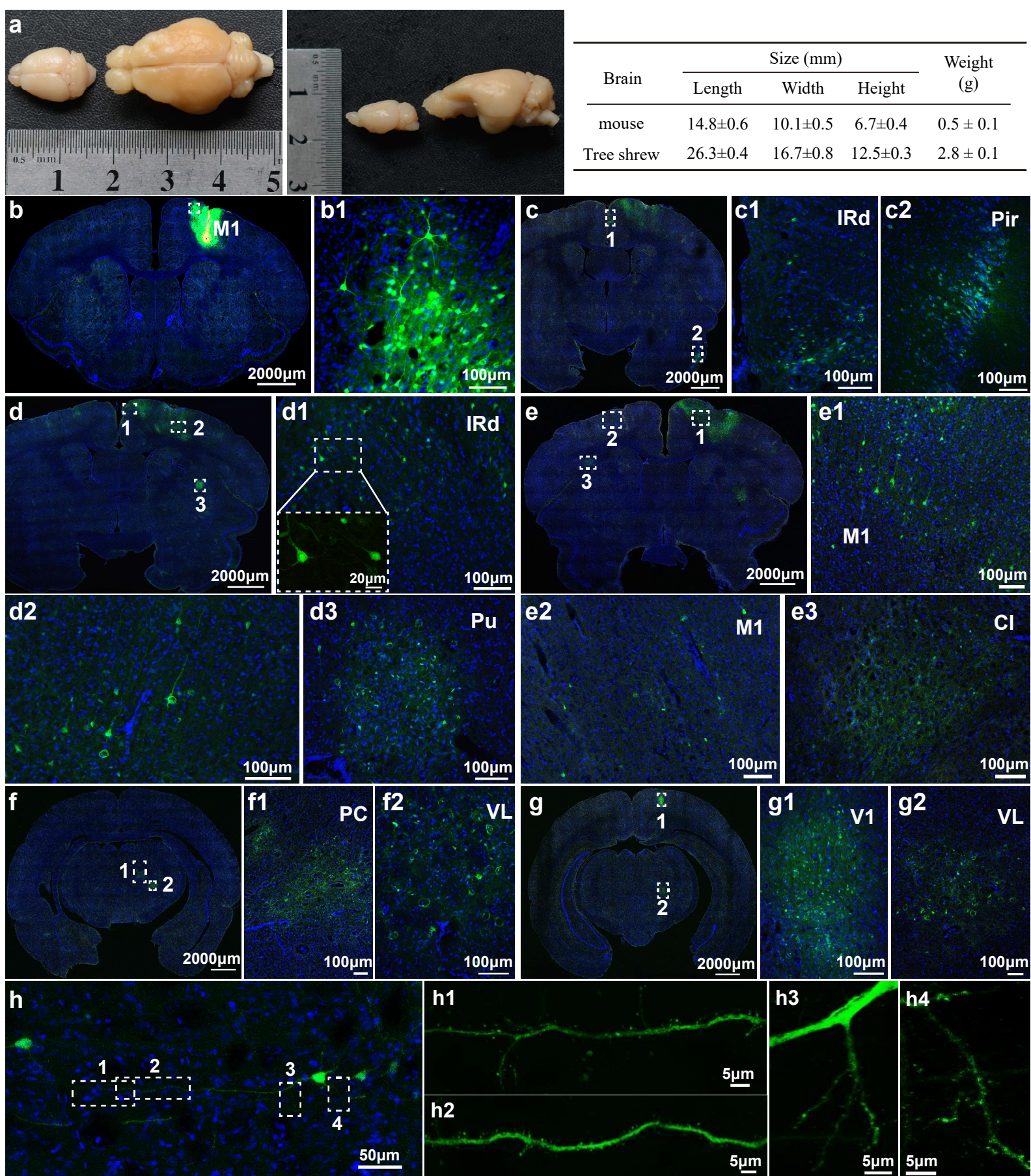

Supplementary Figure 1

Supplement: Supplementary file 1 — Application of H129-G4 in tree shrew (a) Comparison of mouse and tree shrew brains. The brains of adult mouse and tree shrew are imaged with top (left) or side view (middle) after perfusion and fixation. The average size and weight of the brains are presented (right) as mean ± SD (standard deviation) from 5 animals in each group. (b-g) Tracing results H129-G4 in tree threw M1 circuit. H129-G4 and CTB were injected into the M1of adult tree shrews, and the brains were perfused at 6 dpi. Representative images of the coronal brain sections are presented, and the boxed regions are displayed with a higher magnification. M1, primary motor cortex; IRd, infraradiata dorsalis; Pir, piriform cortex; Pu, putamen; Cl, claustrum (Cl); PC, paracentral thalamic nucleus; VL, ventrolateral thalamic nucleus; V1, primary visual cortex. (h) A representative H129-G4 labeled single neuron in tree threw. A representative GFP-labeled neuron around the injection site is shown, and the magnified images of the apical (h1-h3) and basal dendrites (h4) are presented in the right panels. (PDF 3120 kb) [file 13024_2017_179_MOESM1_ESM.pdf]

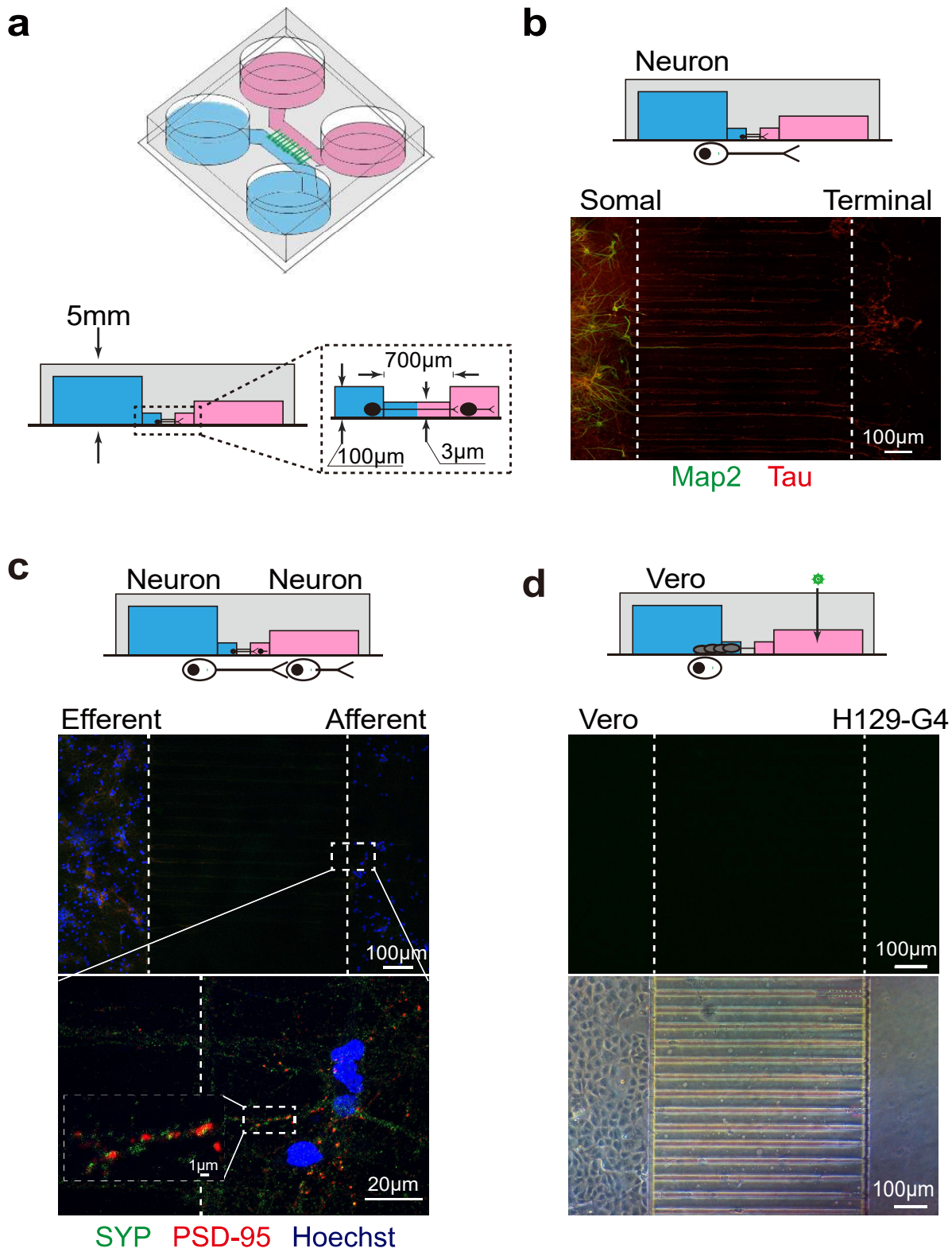

Supplementary Figure 2

Supplement: Supplementary file 2 — The microfluidic plate (a) The schematic structure diagram of the microfluidic system. (b) Axons through the microchannels. Freshly isolated fetal mouse hippocampal and cortical neurons were seeded into one chamber of the microfluidic plate, and cultured for 7 days with positive hydrostatic pressure in the soma chamber. Then the plate was disassembled and stained with antibodies against Tau and Map2. Shown is the representative image from 3 plates. (c) Pre- and post-synaptic markers in the afferent chamber. Neurons were sequentially plated into both chambers at Day 1 and Day 5 respectively, and cultured for additional 7 days with positive hydrostatic pressure in the efferent chamber. The plate was disassembled on Day 12 and stained for pre-synaptic marker synaptophysin (SYP) and post-synaptic marker PSD-95. The nuclei were counterstain with Hoechst dye. Shown is the representative image from 3 plates. (d) No inter-compartment leakage between the chambers. Vero cells were cultured in one chamber with positive hydrostatic pressure, and H129-G4 was added into the opposite chamber to a final concentration of 2.5 × 10^9^ pfu/ml. The GFP signal in the Vero cell culture chamber was monitored, and show is the representative image at 72 hpi. (PDF 676 kb) [file 13024_2017_179_MOESM2_ESM.pdf]

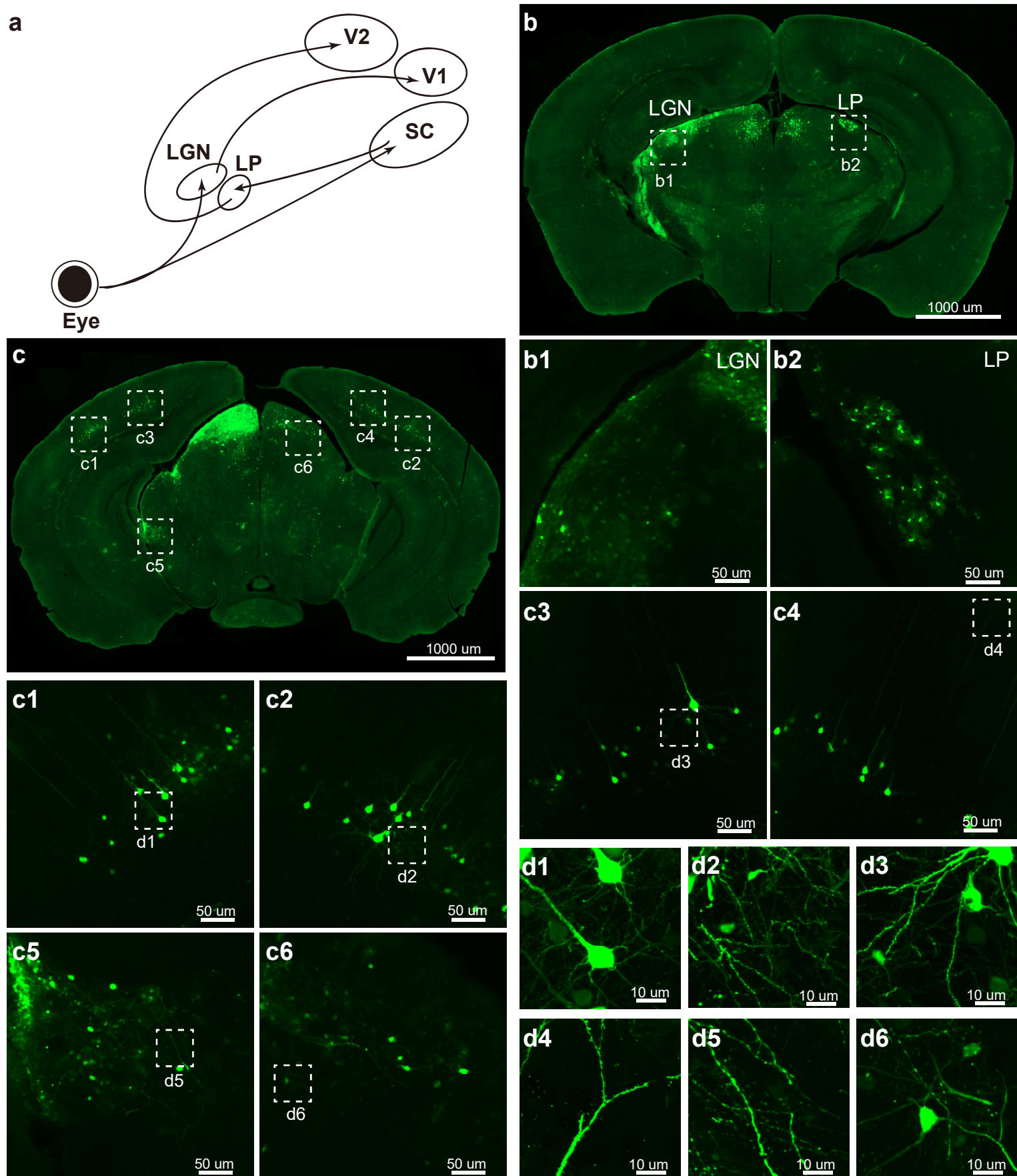

Supplementary Figure 4

Supplement: Supplementary file 4 — Anterograde transmission of H129-G4 from retina to CNS (a) Schema of the simplified mouse visual pathway. LGN, lateral geniculate nucleus; LP, lateral posterior thalamic nucleus; SC, superior colliculus; V1 and V2, primary and secondary visual cortex. (b-d) H129-G4 tracing from retina. H129-G4 was injected into the right retina of wild-type C57BL/6 mice, and images were obtained at 6 dpi. Representative images at LGN, LP (b) and visual cortex (c) are shown. Selected regions are magnified correspondingly, and representative single neurons are presented (d). (PDF 1100 kb) [file 13024_2017_179_MOESM4_ESM.pdf]

**a**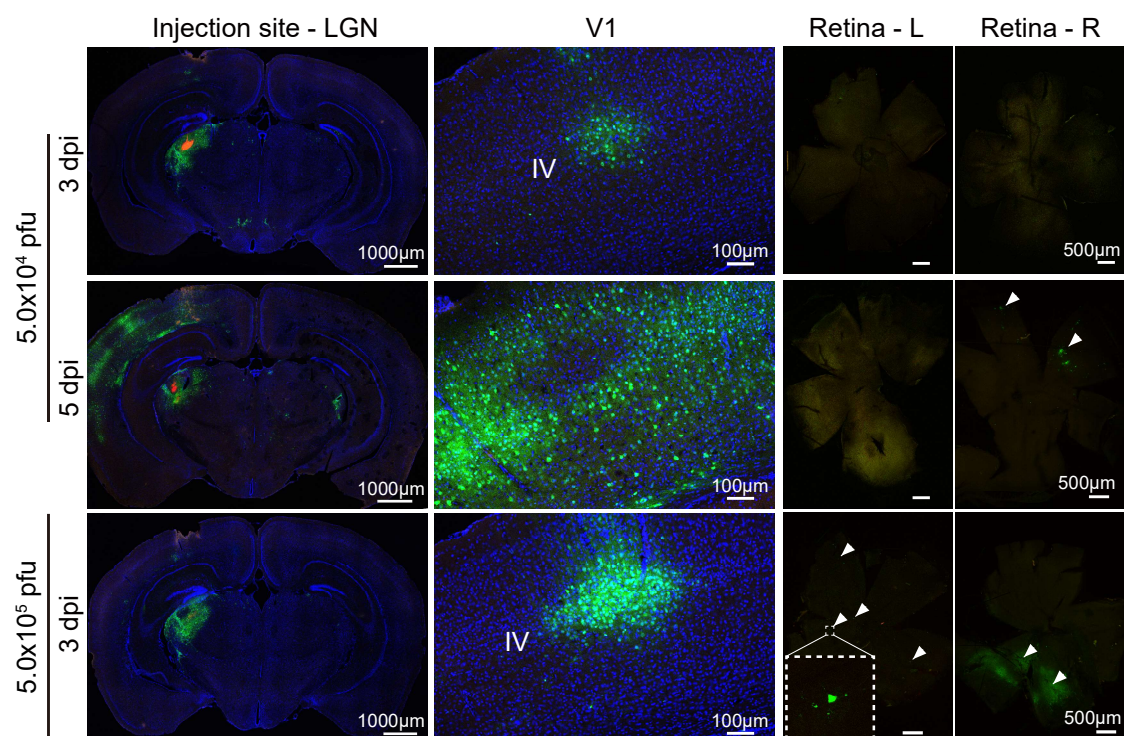**b**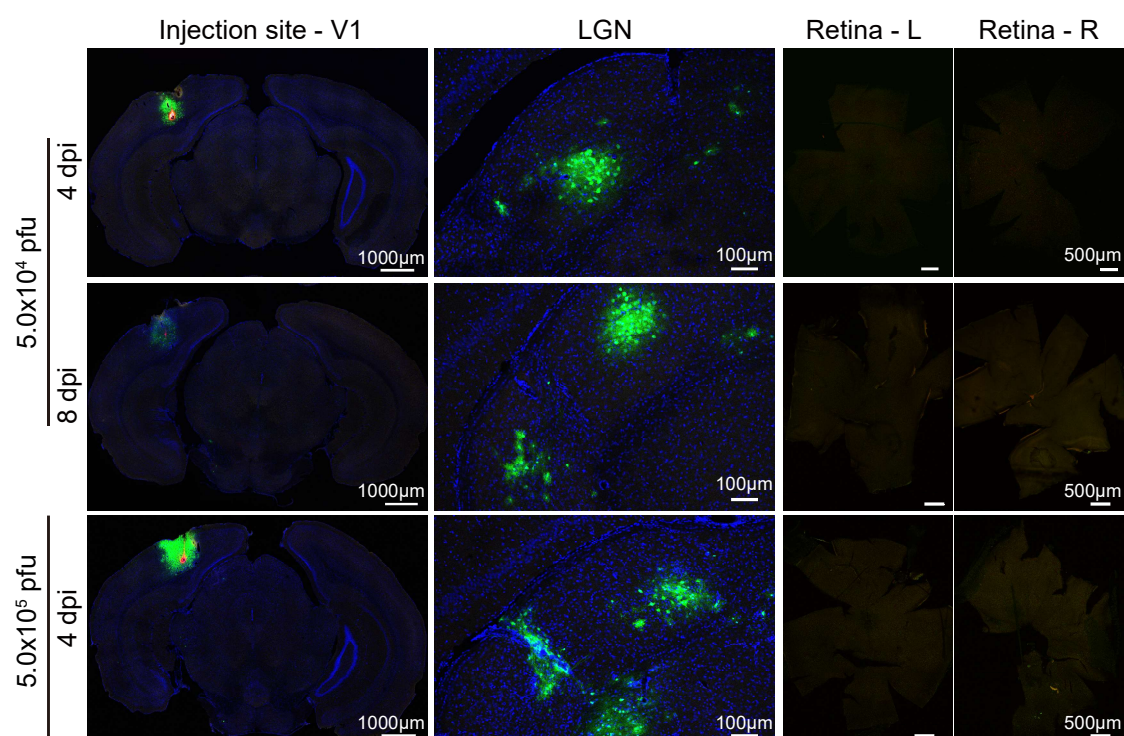

Supplement: Supplementary file 5 — Invasion and transmission of H129-G4 in the visual pathway. Different amount of H129-G4 was injected into the left LGN (a) or V1 (b) of wild-type C57BL/6 mice together with CTB, respectively. The animals were perfused at the indicated time points, and the coronal brain slices throughout the entire brains were observed. Representative images at V1, LGN and retinas are presented. (PDF 1400 kb) [file 13024_2017_179_MOESM5_ESM.pdf]

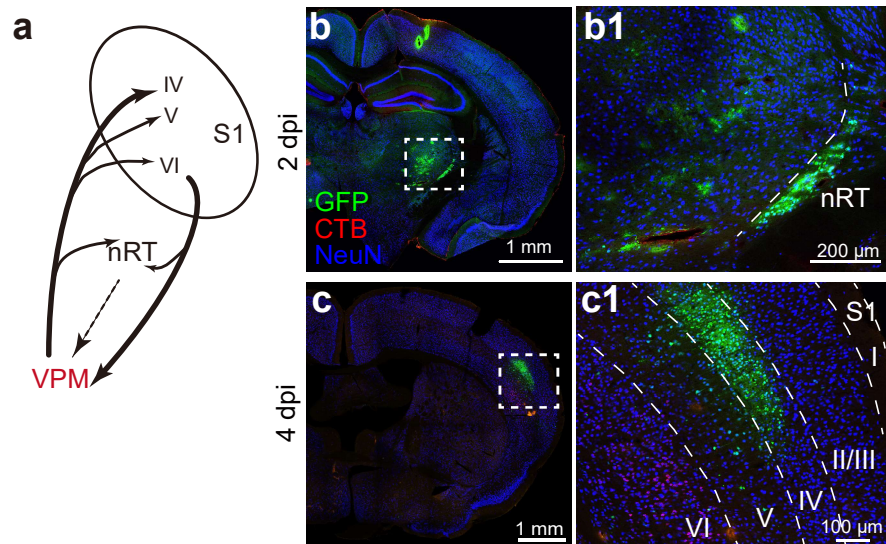

Supplementary Figure 6

Supplement: Supplementary file 6 — Invasion and transmission of H129-G4 in the VPM-S1 circuit. (a) Simplified schema of the VPM-S1 circuit. VPM, ventral posteromedial thalamic nucleus; nRT, nucleus of reticular thalamus; S1, primary somatosensory cortex; IV, V and VI, layer 4, 5 and 6 of the cortex. (b) Representative tracing results of H129-G4 in VPM-S1 circuit. H129-G4 (1 × 10^6^ pfu in 200 nl) was injected into the VPM of wild-type C57BL/6 mice together with Alexa Fluor 594-conjugated CTB (CTB, red). The animals were perfused at the indicated time points, and representative images of the coronal brain slice at the VPM-S1 regions are shown. The boxed areas are magnified and presented in the right panel. The layers of the S1 cortex were determined according to NeuN staining and indicated by the dotted lines. (PDF 503 kb) [file 13024_2017_179_MOESM6_ESM.pdf]

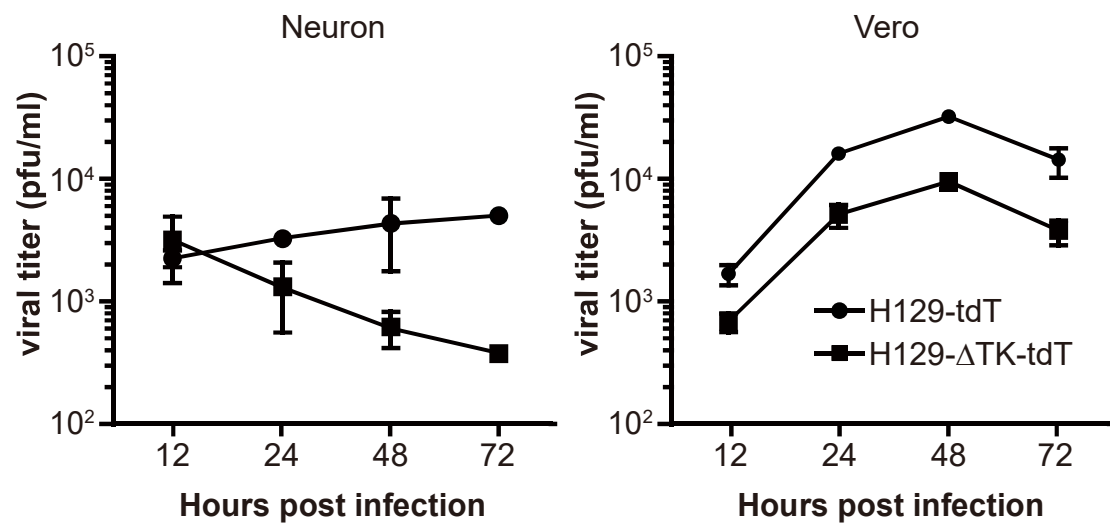

Supplementary Figure 7

Supplement: Supplementary file 7 — In vitro replication of the H129-ΔTK-tdT. To determine the growth property of H129-ΔTK-tdT which lacks TK, fetal mouse hippocampal and cortical neurons (Neuron) or Vero cells were infected with H129-ΔTK-tdT or the TK competent strain H129-tdT at an MOI of 0.02. At the indicated time point, virus titers in the cell culture were determined by standard plaque forming assay. Shown is the representative data from 3 independent experiments, and presented as the Mean ± SD from triplicates. (PDF 183 kb) [file 13024_2017_179_MOESM7_ESM.pdf]

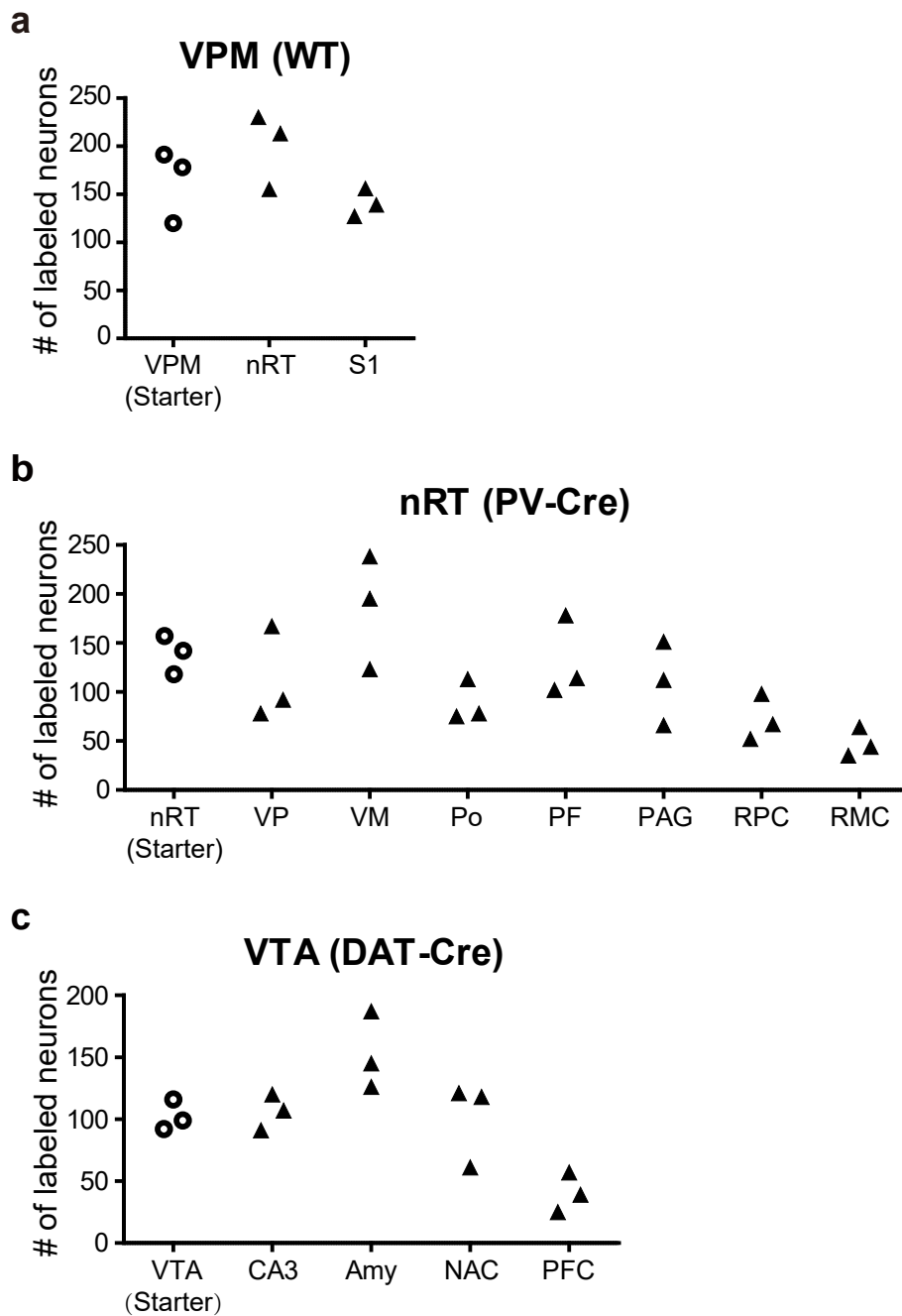

Supplementary Figure 9

Supplement: Supplementary file 9 — Quantitative analysis of H129-ΔTK-tdT monosynaptic tracing. Helper AAVs and H129-ΔTK-tdT were sequentially injected at VPM (a), nRT (b) or VTA (c) of wild type C57BL/6, PV-Cre or DAT-Cre mice respectively, as described in Fig.4-6. The brains’ corona slices were observed with an interval of 120 μM (every 4 slices), and the amounts of the labeled cell at the indicated brain regions were counted. The starter cells at the injection site were detected at Day32 as tdTomato and GFP co-expressing neurons (opened circle, each circle represents one mouse), and the tdTomato positive neurons at other brain regions were observed at Day 32 (filled triangle, each circle represents one mouse). (PDF 109 kb) [file 13024_2017_179_MOESM9_ESM.pdf]

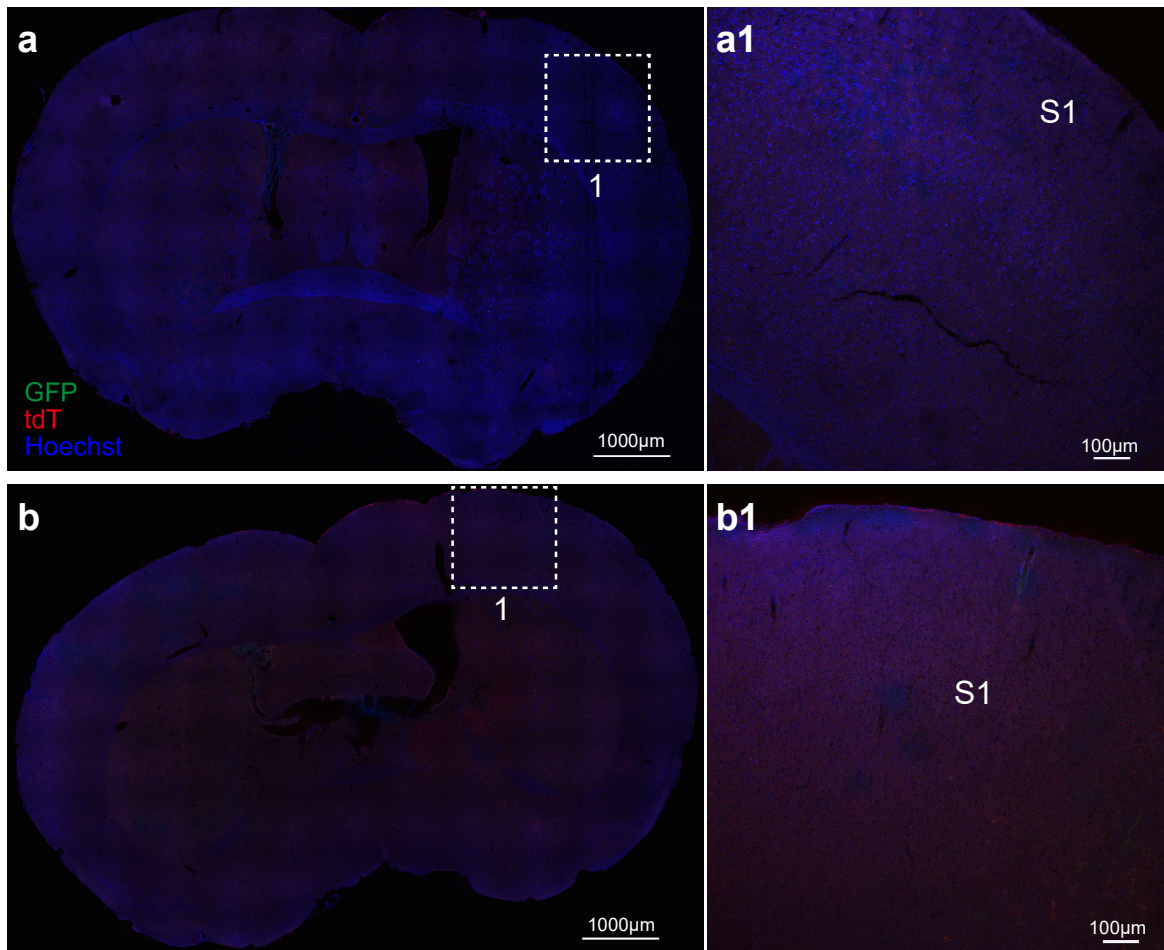

Supplementary Figure 10

Supplement: Supplementary file 10 — Absence of labeled cells upstream of nRT-PV neurons. H129-ΔTK-tdT and AAV-DIO-TK-GFP were injected into the nRT of PV-Cre mice as shown in Fig.5. The animals were perfused Day 32, and the coronal brain slices throughout the entire brains were observed. The representative images of S1, the upstream region of nRT, are shown. (PDF 929 kb) [file 13024_2017_179_MOESM10_ESM.pdf]
