# Supplementary material for: Anterograde monosynaptic transneuronal tracers derived from herpes simplex virus 1 strain H129
Source: Mol Neurodegener. 2017 May 12;12:38. doi: 10.1186/s13024-017-0179-7 (PMC5427628; doi:10.1186/s13024-017-0179-7)
Supplement: Supplementary file 3 — Terminal invasion of H129-G4 in vitro (a) Dose related incidence of H129-G4 terminal invasion. Fetal mouse hippocampal and cortical neurons were cultured as described above, H129-G4 was added to the terminal chamber at the indicated final concentrations, and GFP signal was monitored daily. Representative images at 48 hpi from 3 plates at each concentration group are shown. The labeled neurons are indicated with the dotted boxes and magnified in the lower panels. Scale bar = 100 μm. (b) Census of H129-G4 terminal invasion. Hippocampal and cortical neurons, trigeminal ganglion (TG) and dorsal root ganglion neurons (DRG) were cultured in microfluidic plates at the indicated cell amount. H129-G4 was added to the axonal terminal chamber at different final concentrations, and the GFP positive neurons in each plate were counted. Data were from 3 plates under each condition, and results are presented as mean ± SD. (c) Terminal invasion of VSV. Similarly, VSV-GFP was added into the terminal chamber of hippocampal and cortical neurons to a final concentration of 1 × 10^7^ pfu/ml. The representative image from 3 plates at 48 hpi is shown. Scale bar = 100 μm. (PDF 429 kb) [file 13024_2017_179_MOESM3_ESM.pdf]

a

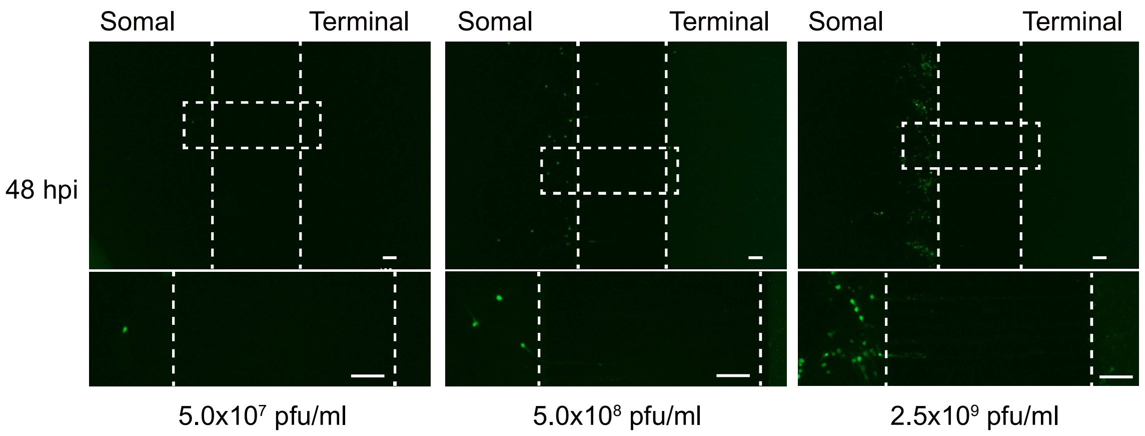

b

Terminal invasion in different conditions

Neuron type	Virus concentration (pfu/ml)	Labelled neuron amount		
		24 hpi	48 hpi	72 hpi
Hipp. and cortical neuron 5x10^5 cells	1.0x10^7	0	0	0
	5.0x10^7	0	0.3 ± 0.5	0.3 ± 0.5
	5.0x10^8	0	1.3 ± 1.2	1.3 ± 1.2
	2.5x10^9	6.7 ± 4.5	72.0 ± 8.0	89.0 ± 12.0
Trigeminal ganglion 1x10^5 cells	1.0x10^7	0	0	0
	5.0x10^7	0	4.3 ± 1.7	4.3 ± 1.7
	5.0x10^8	5.0 ± 3.6	9.7 ± 2.1	16.0 ± 3.6
	2.5x10^9	10.7 ± 6.6	44.0 ± 7.0	97.3 ± 26.0
Dorsal root ganglion 1x10^5 cells	1.0x10^7	0	0	0
	5.0x10^7	0	4.7 ± 3.7	6.0 ± 2.2
	5.0x10^8	1.0 ± 1.4	8.0 ± 5.1	9.3 ± 5.0
	2.5x10^9	26.7 ± 11.3	51.0 ± 13.1	75.3 ± 15.1

c

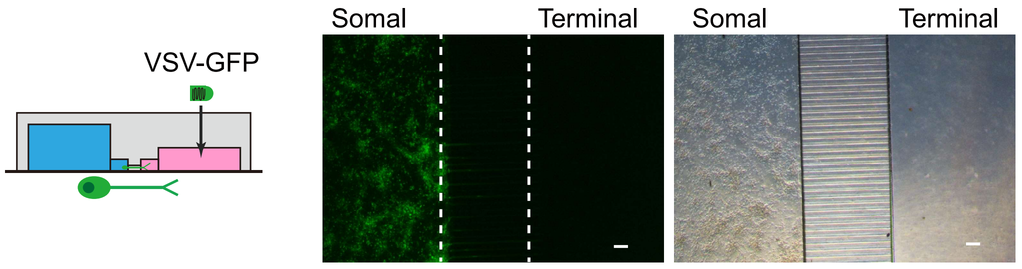

Supplementary Figure 3
