# Supplementary material for: Anterograde monosynaptic transneuronal tracers derived from herpes simplex virus 1 strain H129
Source: Mol Neurodegener. 2017 May 12;12:38. doi: 10.1186/s13024-017-0179-7 (PMC5427628; doi:10.1186/s13024-017-0179-7)
Supplement: Supplementary file 8 — H129-ΔTK-tdT alone in various brain regions. (a) Parameters for H129-ΔTK-tdT injection. (b-g) Representative labeling results. H129-ΔTK-tdT was injected into the indicated brain regions of wild type C57BL/6 as listed in the table (a), and the brains were perfused at 10 dpi. The coronal brain slice throughout the brain were observed after staining for NeuN (blue), GFAP (green) and tdTomato (red). Representative images of upstream and downstream region of the injection sites are shown. The white arrows indicated the injection sites. The neurons in the upstream brain region labeled by virus terminal invasion are indicated by the white arrowheads (e3). OB, olfactory bulb; Pir, piriform cortex; MOE, main olfactory epithelium; M1, primary motor cortex; DG, dentate gyrus; LEC, lateral entorhinal cortex; LGN, lateral geniculate nucleus; V1, primary visual cortex; VTA, ventral tegmental area; LDTg, laterodorsal tegmental nucleus. (PDF 2370 kb) [file 13024_2017_179_MOESM8_ESM.pdf]

a

H129-ΔTK-tdT alone in various brain regions

Nucleus	Coordinates (mm)			H129-ΔTK-tdT		Animal amount
	ML	AP	DV	Dose (pfu)	volume (μl)	
OB	-0.48	+4.28	-2.30	1.75×10^5	0.35	3
M1	-1.70	+1.54	-1.75	1.0×10^5	0.2	3
DG	-1.25	-2.06	-2.00	7.5×10^4	0.15	3
CA1	-1.30	-2.18	-1.50	1.0×10^5	0.2	3
LGN	-2.13	-2.30	-2.75	1.0×10^5	0.2	3
VTA	-0.40	-3.28	-4.30	1.0×10^5	0.2	3

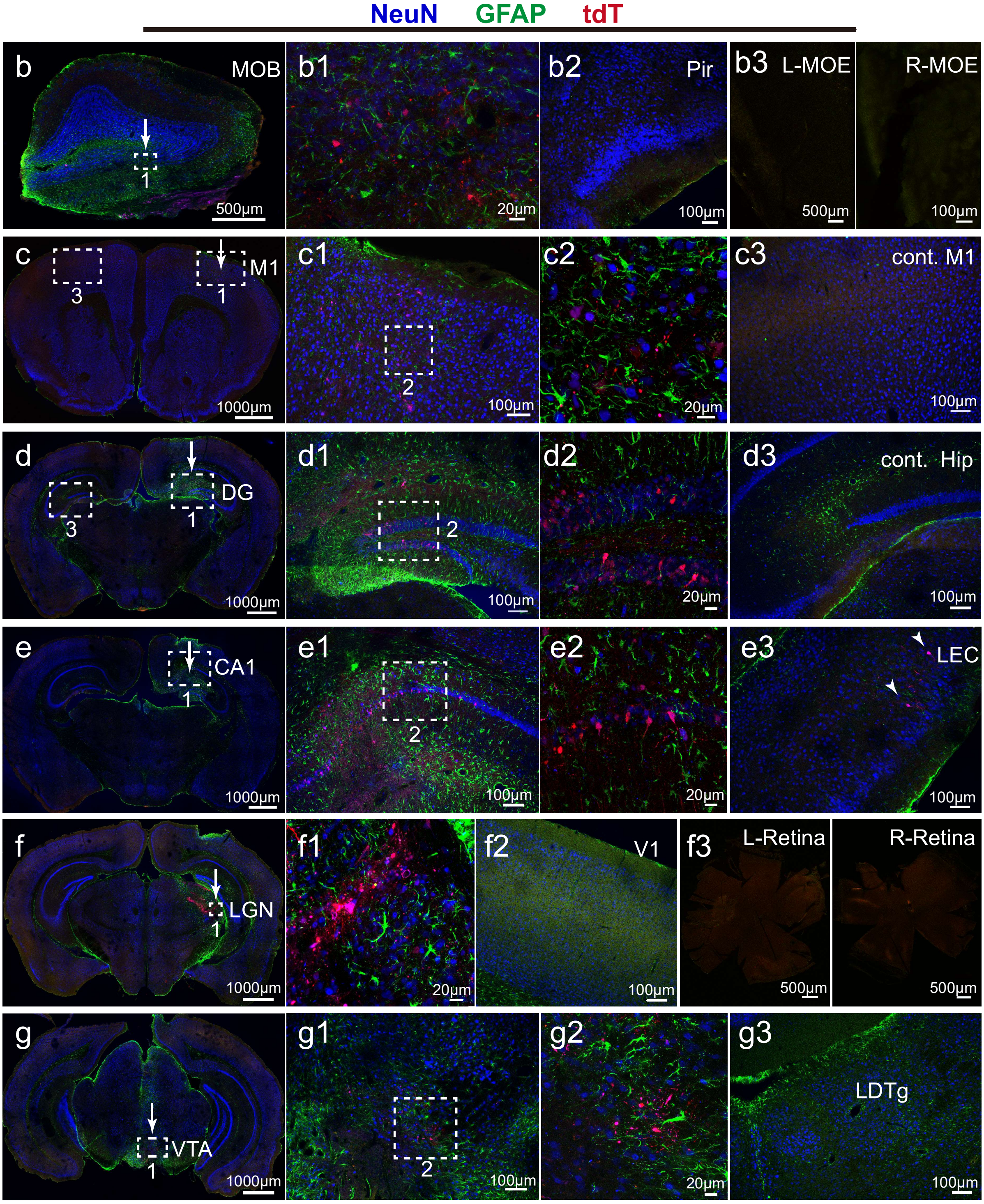

Supplementary Figure 8
